# *Lactobacillus paracasei*-derived extracellular vesicles reverse molecular and behavioral deficits in mouse models of autism spectrum disorder

**DOI:** 10.1038/s12276-025-01429-w

**Published:** 2025-04-01

**Authors:** Jin-Young Park, Eun-Hwa Lee, Ji-Eun Kim, Jae-Won Paeng, Jin-Chul Paeng, Tae-Kyung Kim, Yoon-Keun Kim, Pyung-Lim Han

**Affiliations:** 1 Department of Brain and Cognitive Sciences, Scranton College, Ewha Womans University, Seoul, Republic of Korea; 2 Department of Physical Education and Sport Science Institute, Korea National Sport University, Seoul, Republic of Korea; 3 MD Healthcare Inc., Seoul, Republic of Korea; 4 Department of Nuclear Medicine, Seoul National University Hospital, Seoul, Republic of Korea

**Keywords:** Autism spectrum disorders, Autism spectrum disorders

## Abstract

Autism spectrum disorder (ASD) is a heterogeneous group of neurodevelopmental disorders characterized by social communication deficits and repetitive behaviors. Although our current understanding the mechanisms underlying ASD is growing, effective treatment options are still underdevelopment. Extracellular vesicles derived from the probiotic *Lactobacillus paracasei* (LpEV) have shown neuroprotective effects in both in vitro and in vivo models. Here we investigate whether LpEV can alleviate core symptoms in genetic ASD models that exhibit accumulated developmental deficits. Dopamine receptor D2 (Drd2)-knockout (KO) mice exhibit social behavior deficits and excessive grooming, core symptoms of ASD. LpEV treatment significantly improves these autistic-like behaviors in Drd2-KO mice, suggesting that LpEVs can mitigate the persistent dysregulation of signaling pathways in these mice. RNA sequencing followed by Gene Ontology enrichment analysis of LpEV-treated Drd2-KO mice identifies distinct groups of genes altered in the brain of Drd2-KO mice, which were reversed by LpEV treatment. Notably, a high proportion of these genes overlap significantly with known ASD genes in the SFARI database, strengthening the potential of LpEV to target relevant pathways in ASD. Further investigation identifies oxytocin and oxytocin receptor (Oxtr) as potential therapeutic targets. LpEV treatment significantly improves autistic-like behaviors in Oxtr-KO heterozygous mice, adenylyl cyclase-5 KO mice and Shank3-KO mice, suggesting its therapeutic potential to target ASD through broader mechanisms beyond a single gene pathway. These results highlight the therapeutic potential of LpEV in reversing the accumulated dysregulated signaling pathways leading to ASD symptoms and improving autistic-like behaviors.

## Probiotic-derived nanovesicles restore behavioral deficits in autism

Autism spectrum disorder (ASD) is a condition that affects social interaction and repetitive behaviors. Researchers studied the effects of extracellular vesicles (EVs) from *Lactobacillus paracasei*, a type of probiotic bacteria, on genetic mouse models of ASD. EVs are tiny particles released by cells that can carry proteins, nucleic acids and other molecules. The study involved treating different genetic models of mice with these EVs to see if they could improve ASD-like symptoms. They found that the EVs improved social behaviors and reduced repetitive actions in the mice. They also discovered changes in the expression of genes closely related to ASD in the brain, suggesting that the EVs might help to correct some of the underlying molecular issues. These findings suggest that *Lactobacillus paracasei*-derived EVs could be a promising new approach for treating ASD symptoms.

This summary was initially drafted using artificial intelligence, then revised and fact-checked by the author.

## Introduction

Autism spectrum disorder (ASD) is a neurodevelopmental disorder characterized by social communication difficulties and repetitive behaviors^1^. This dysfunction typically begins early in childhood. It is thought to arise from a complex interplay of genetic and environmental factors that accumulate during neural development. Over 1200 genes or genetic loci linked to ASD have been identified, affecting various neuronal functions, including synapse formation, gene expression regulation and cellular signaling in neural development^2,3^. In addition, diverse environmental factors such as maternal stress or infection during pregnancy and gut microbiota dysbiosis may also increase the risk of ASD^4–6^. The etiological heterogeneity is further compounded by developmentally accumulated dysregulation in brain regions, presenting a notable challenge in developing effective treatments for people with diverse causes of ASD. This complexity in both development and underlying causes necessitates the use of diverse models to dissect the pathophysiology of ASD and the development of multiple treatment options beyond a single pathway.

Mounting evidence indicates that dysfunction in the striatum, a brain region involved in movement, reward and stress, contributes to the development of core ASD symptoms^7,8^. An earlier study reported that mice lacking Shank3, a postsynaptic scaffold protein with preferential expression in the striatum, had defective excitability of striatal neurons and exhibited autistic-like behaviors^9^. Subsequent genetic or pharmacological studies demonstrated that an excessive increase of dopamine inputs into the dorsal striatum (dStr) induces social behavior deficits and excessive grooming. Dopamine receptor D2 (Drd2) is preferentially expressed in the striatum, hippocampus and frontal cortex^10^. Drd2-knockout (KO) mice display autistic-like behaviors^11^. Furthermore, siRNA-mediated knockdown of *Drd2*, *GluN2B*, *GluA1* or *mGluR3* within the dStr produced substantial deficits in social behaviors and excessive grooming^11–13^. Adenylyl cyclase-5 (Adcy5) is highly expressed in the striatum and hippocampus, where it converts the activation of various G-protein-coupled receptors, including D2 dopamine receptors, opioid receptors and metabotropic glutamate receptor 5 (mGluR5), into intracellular signals^12,14,15^. Adcy5-KO mice also exhibit autistic-like behaviors^12^. Consistently, clinical evidence indicates a structural alteration in the striatum or an enhanced diffused connectivity between the striatum and cortical areas in patients with ASD^16–18^. Regarding that the striatum and its associated neural circuits play a crucial role in regulating ASD core symptoms, mouse models that target genes regulating these brain regions hold promise as a valuable first step in developing treatment options for core ASD symptoms.

Recent studies have reported that extracellular vesicles (EVs) derived from Gram-positive and Gram-negative bacteria confer beneficial effects on various disease models, from those affecting the periphery to those of the brain. EVs derived from *Lactobacillus plantanum*, *Bacillus subtilis* and *Akkermansia muciniphila* exert therapeutic effects on stress-induced depression through the upregulation of MeCP2, Sirt1 and neurotrophic factors in hippocampal neurons^19,20^. *Lactobacillus paracasei*-derived EVs (LpEVs) produced anti-inflammatory effects against intestinal inflammatory responses in colitis induced by dextran sulfate sodium^21^. Furthermore, LpEV treatment counteracted glucocorticoid-induced genome-wide transcriptional changes in HT22 neuronal cells, rescued stress-induced depression in mice^22^ and ameliorated Aβ pathology and cognitive deficits in Tg-APP/PS1 mice^23^. These results suggest that LpEV has a wide range of therapeutic potentials to restore molecular and cellular dysfunction experimentally induced in adult animals. However, it has not been tested whether LpEV or any other specific bacterial EV can improve developmentally originated neuropathology caused by genetic mutations, such as that seen in ASD.

This study investigated whether LpEV could alleviate both molecular and behavioral deficits in genetic ASD models. This study demonstrated that LpEV treatment reversed the dysregulated signaling pathways important for ASD-associated neuronal function and improved autistic-like behaviors.

## Materials and methods

The details of the methods and materials are provided in the Supplementary Information.

### Animals

Drd2-KO, AC5-KO, Oxtr-KO and Shank3-KO mice were described previously^9,12,24–26^. All mice were handled in accordance with the Guidelines of Animal Care at Ewha Womans University through the permission of the International Animal Care and Use Committee of Ewha Womans University (no. 19-015).

### Preparation of EVs from *Lactobacillus paracasei* culture

*Lactobacillus paracasei* culture and EV isolation were described previously^21,22^.

## Results

### Behavioral readouts and molecular biomarkers for autism-like phenotypes in Drd2-KO mice

Drd2-KO mice exhibit social behavior deficits and repetitive behaviors^11^. We hypothesized that these behavioral impairments stem from the combined effects of Drd2 deficiency and disrupted downstream signaling pathways during brain development. To evaluate LpEV’s therapeutic potential in this model, we examined behavioral readouts and relevant molecular markers in Drd2-KO mice.

**Fig. 1:**
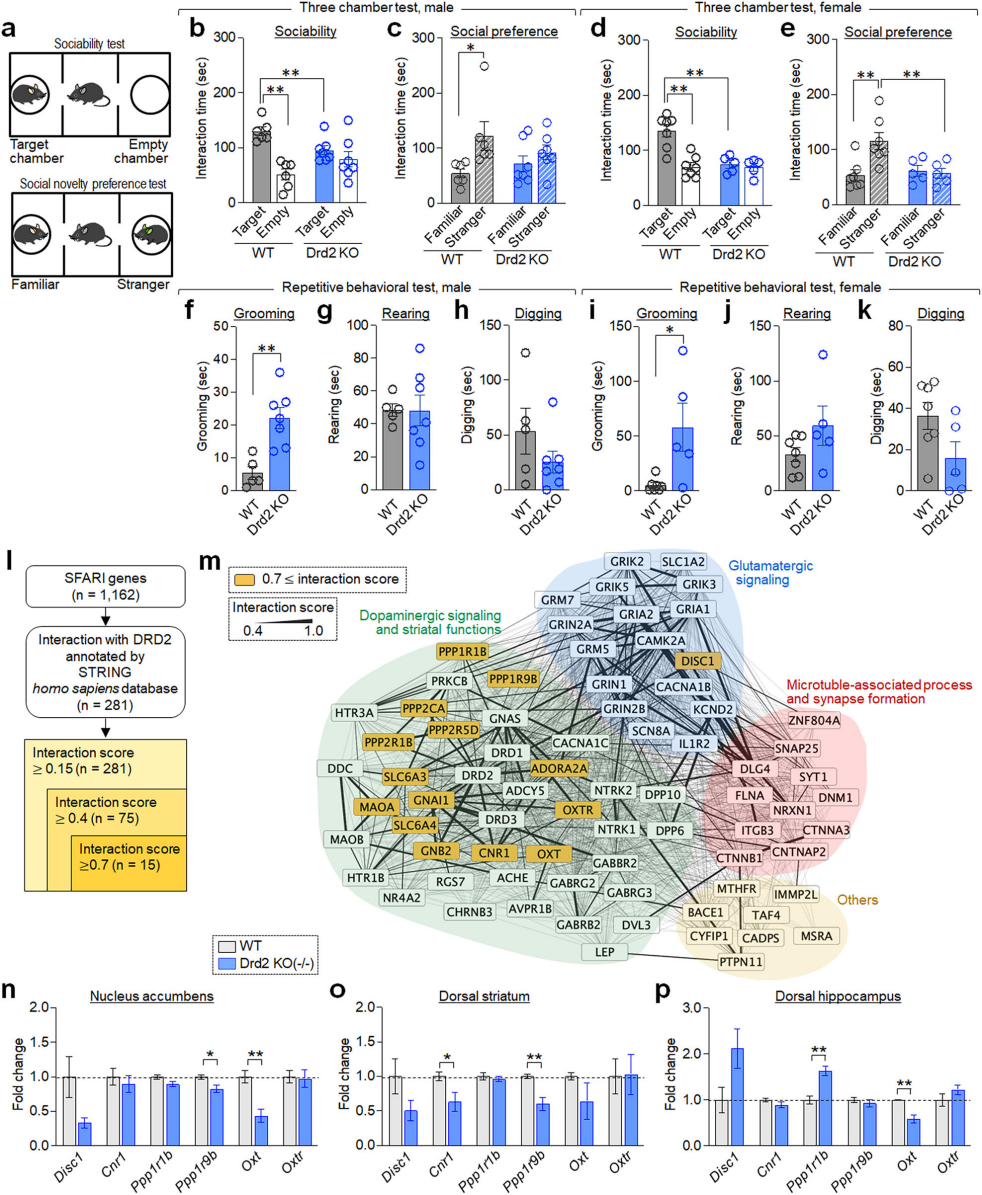
Behavioral and molecular biomarkers for autism-like phenotypes in Drd2-KO mice. **a**, Layouts of the three-chamber apparatus for testing sociability (top) and social novelty preference (bottom). **b**–**e**, Social behaviors in Drd2-KO mice: time spent exploring a social target compared with an empty cage (sociability; **b** and **d**) and time spent exploring a novel stranger versus a familiar one (social novelty preference; **c** and **e**) in the three-chamber social behavior test. Data shown for both male (**b** and **c**) and female (**d** and **e**) mice. Male: wild type (WT), 6 animals; Drd2-KO, 7 animals. Female: WT, 7 animals; Drd2-KO, 5 animals. Male (**b** and **c**). Sociability (**b**). WT versus Drd2-KO. Two-way analysis of variance (ANOVA), Fisher’s least significant difference (LSD) post-hoc test. Genotype, *F*(1,22) = 0.09477, *P* = 0.7611; Social target, *F*(1,22) = 19.56, *P* = 0.0002; genotype × social target, *F*(1,22) = 8.379, *P* = 0.0084. Social preference (**c**). WT versus Drd2-KO. Two-way ANOVA, Fisher’s LSD post-hoc test. Genotype, *F*(1,22) = 0.1337, *P* = 0.7182; social target, *F*(1,22) = 7.112, *P* = 0.0141; genotype × social target, *F*(1,22) = 2.033, *P* = 0.1680. Female (**d** and **e**). Sociability (**d**). WT versus Drd2-KO. Two-way ANOVA, Fisher’s LSD post-hoc test. Genotype, *F*(1,20) = 11.43, *P* = 0.0030; social target, *F*(1,20) = 15.11, *P* = 0.0009; genotype × social target, *F*(1,20) = 10.98, *P* = 0.0035. Social preference (**e**). WT versus Drd2-KO. Two-way ANOVA, Fisher’s LSD post-hoc test. Genotype, *F*(1,20) = 4.164, *P* = 0.0547; social target, *F*(1,20) = 5.396, *P* = 0.0308; genotype × social target, *F*(1,20) = 7.249, *P* = 0.0140. **f**–**k**, Self-directed repetitive behaviors. Time spent by male and female Drd2-KO mice on grooming (**f** and **i**), rearing (**g** and **j**) and digging (**h** and **k**). Male: WT, 5 animals; Drd2-KO, 7 animals. Female: WT, 7 animals; Drd2-KO, 5 animals. Male (**f**–**h**). WT versus Drd2-KO. Student’s *t*-test. Grooming, *t* = 4.012, *P* = 0.0025; rearing, *t* = 0.03921, *P* = 0.9695; digging, *t* = 1.334, *P* = 0.2119. Female (**i**–**k**). WT versus Drd2-KO. Student’s *t*-test. Grooming, *t* = 2.890, *P* = 0.0161; rearing, *t* = 1.605, *P* = 0.1396; digging, *t* = 1.987, *P* = 0.0749. **l**,**m**, Drd2-interacting genes identified from 1,162 SFARI genes; 281 genes at confidence scores ≥0.15; 75 genes at confidence scores ≥0.4; 15 genes at confidence scores ≥0.7 (**l**). Protein–protein interaction networks with four functional groups grouped based on their possible BPs (**m**). **n**–**p**, RT-PCR data showing changes in expression levels of *Disc1*, *Cnr1*, *Ppp1r1b (DARPP-32)*, *Ppp2r1b*, *Oxt* and *Oxtr* in the dStr (**n**), NAc (**o**) and dHP (**p**) of Drd2-KO mice. *n* = 4 animals per group. Four PCR repeats per group. WT versus Drd2-KO. Student’s *t*-test. dStr (**n**): Disc1, *t* = 2.190, *P* = 0.0711; Cnr1, *t* = 0.6145, *P* = 0.5615; Ppp1r1b, *t* = 2214, *P* = 0.0688; Ppp1r9b, *t* = 2.845, *P* = 0.0294; Oxt, *t* = 4.369, *P* = 0.0047; Oxtr, *t* = 0.1649, *P* = 0.8744. NAc (**o**): Disc1, *t* = 1.669, *P* = 0.1462; Cnr1, *t* = 2.452, *P* = 0.0496; Ppp1r1b, *t* = 0.6174, *P* = 0.5597; Ppp1r9b, *t* = 4.007, *P* = 0.0071; Oxt, *t* = 1.352, *P* = 0.2250; Oxtr, *t* = 0.07638, *P* = 0.9416. dHP (**p**): Disc1, *t* = 2.209, *P* = 0.0693; Cnr1, *t* = 1.456, *P* = 0.1956; Ppp1r1b, *t* = 4.660, *P* = 0.0035; Ppp1r9b, *t* = 0.7361, *P* = 0.4894; Oxt, *t* = 4.983, *P* = 0.0025; Oxtr, *t* = 1.290, *P* = 0.2444.

In the three-chamber sociability test, wild-type mice spent significantly more time interacting with a social target over an empty cage. Conversely, Drd2-KO mice spent a similar amount of time with the social target and the empty cage in both males and females. In the following social novelty preference test, wild-type mice spent more time with the stranger compared with the familiar one. However, Drd2-KO mice again showed no significant difference in time spent with the stranger or the familiar mouse, confirming deficits in sociability and social novelty preference in both males and females (Fig. 1a–e). In the repetitive behavior tests, both male and female Drd2-KO mice displayed significantly increased self-grooming, suggesting its potential as a valuable measure of repetitive behavior. However, no significant differences in rearing and digging behaviors were found between the two groups (Fig. 1f–k).

To identify potential molecular biomarkers underlying autistic-like behaviors in Drd2-KO mice, we investigated Drd2-associated functional players from the Simons Foundation Autism Research Initiative (SFARI) gene database, a comprehensive resource that compiles ASD genes. As of 16 January 2024, it released 1162 genes. We performed Gene Ontology (GO) enrichment analysis on these genes to identify genes interacting with Drd2 using the STRING database. This analysis identified 75 genes potentially interacting with Drd2 (confidence score ≥0.4). These genes could be categorized into four main functional groups based on their associated biological processes (BPs): (1) dopamine signaling and striatal function (40 genes); (2) glutamatergic signaling (17 genes); (3) microtubule-associated process and synapse formation (11 genes); and (4) others (unclassified; 8 genes) (Fig. 1l, m). Among these, 15 genes, including SLC6A3 (dopamine transporter), SLC6A4 (serotonin transporter), GNAI1 (G-protein subunit i1), ADORA2A (adenosine A2A receptor), CNR1 (cannabinoid receptor 1), DISC1 (disrupted in schizophrenia 1), PPP2CA (phosphatase 2A catalytic subunit α), PPP2R5D (phosphatase 2 regulatory subunit 5δ), PPP2R1B (phosphatase 2 scaffold subunit Aβ), PPP1R1B (phosphatase 1 regulatory subunit 1B), PPP1R9B (phosphatase 1 regulatory subunit 9B), OXT (oxytocin), OXTR (oxytocin receptor), GNB2 (G protein subunit β 2) and MAOA (monoamine oxidase A) strongly interacted with Drd2 (interaction score ≥0.7) (Fig. 1l, m).

Real-time (RT) PCR analysis in Drd2-KO mice revealed altered expression of ASD-associated genes, *Disc1*, *Cnr1*, *Ppp1r1b* (*DARPP-32*), *Ppp2r1b*, *Oxt* and its receptor *Oxtr*, in the dStr, nucleus accumbens (NAc) and/or dorsal hippocampus (dHP) in Drd2-KO mice. These results suggest that Drd2 deficiency disrupts the expression of ASD-linked genes (Fig. 1n–p).

### Assessment of biodistribution of orally administered LpEV in the body

We investigated the biodistribution of orally administered LpEV that was labeled with radioactive isotope indium (^111^In) via a linker in mice. Mice were administered ^111^In-labeled LpEV, ^111^In-NOTA-PEG_3_-N_3_-LpEV. The radioactivity signals for the isotope-labeled LpEV was located mainly in the stomach and intestines after 1 h. This signal remained significantly in these regions even after 24 h, although at lower levels. By 48 h, most radioactivity disappeared from the body. Further analysis using a gamma counter revealed LpEV distribution beyond the stomach and intestines. The radioactivity was detected in various organs, including the liver, heart, lungs, spleen, kidneys and blood at moderate levels, and even the brain and bone at lower levels (Supplementary Fig. 1). This suggests LpEV is absorbed from the digestive tract followed by distribution throughout the body.

### LpEV treatment improved social behavior deficits and excessive grooming in Drd2-KO mice

**Fig. 2:**
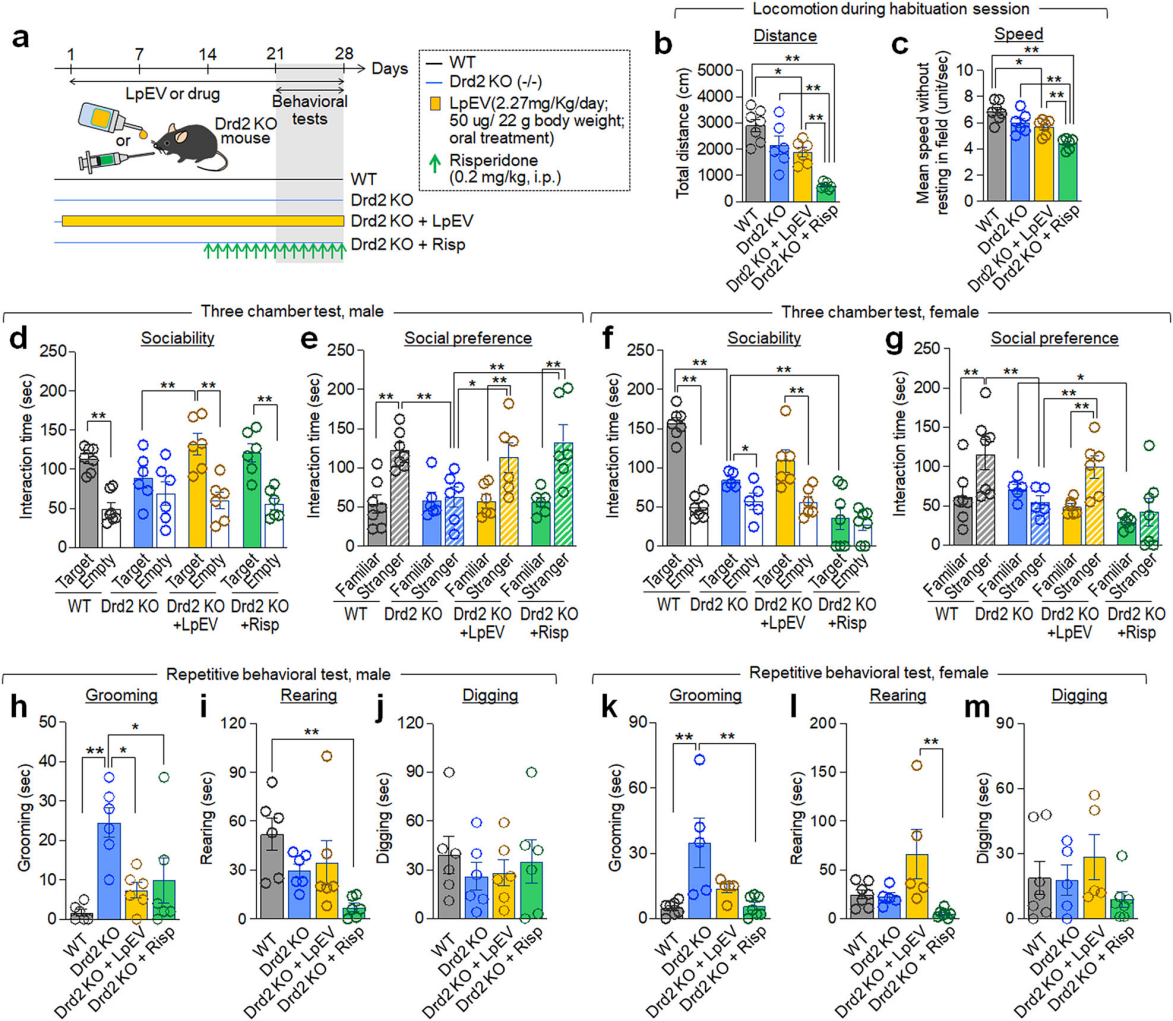
LpEV treatment improved behavioral deficits of Drd2-KO mice. **a**, Experimental design. Drd2-KO mice received LpEV (2.27 mg/kg/day or 1.753 × 10^12^ particles/kg/day, or 50 μg per 22 g body weight per day) orally for 3 weeks and risperidone (0.2 mg/kg/day) was intraperitoneally (i.p.) administered for 7 days, and both treatments continued during behavioral testing as depicted. Behavioral tests were conducted sequentially: social interaction, social preference and repetitive tests. **b**,**c**, Locomotion distance (**b**) and speed (**c**) of WT, Drd2-KO, Drd2-KO+ LpEV and Drd2-KO + risperidone during the habituation period of the three-chamber social behavior test. All males: WT, 7 animals; Drd2-KO, 6 animals; Drd2-KO + LpEV, 6 animals; Drd2-KO + Risp, 6 animals. Total distance (**b**), WT versus Drd2-KO versus Drd2-KO + LpEV versus Drd2-KO + Risp. One-way ANOVA, Tukey’s post-hoc test. *F*(3,21) = 15.49, *P* < 0.0001. Speed (**c**), WT versus Drd2-KO versus Drd2-KO + LpEV versus Drd2-KO + Risp. One-way ANOVA, Tukey’s post-hoc test. *F*(3,21) = 14.64, *P* < 0.0001. **d**–**g**, Social behaviors. Time spent exploring a social target compared to an empty cage (**d** and **f**) and time spent exploring a novel stranger versus a familiar one (**e** and **g**) in the three-chamber social behavior tests. Data shown for male (**d** and **e**) and female (**f** and **g**) Drd2-KO mice. Male: WT, 7 animals; Drd2-KO, 6 animals; Drd2-KO + LpEV, 6 animals; and Drd2-KO + risperidone, 6 animals. Female: WT, 7 animals; Drd2-KO, 5 animals; Drd2-KO + LpEV, 6 animals; and Drd2-KO + risperidone, 7 animals. Male (**d** and **e**). Sociability (**d**). WT versus Drd2-KO. Two-way ANOVA, Fisher’s LSD post-hoc test. Genotype, *F*(1,22) = 0.0504, *P* = 0.8244; social target, *F*(1,22) = 16.12, *P* = 0.0006; genotype × social target, *F*(1,22) = 4.344, *P* = 0.0490. Drd2-KO versus Drd2-KO + LpEV. Two-way ANOVA, Fisher’s LSD post-hoc test, LpEV, *F*(1,20) = 1.678, *P* = 0.2100; social target, *F*(1,20) = 12.28, *P* = 0.0022; LpEV × social target, *F*(1,20) = 3.500, *P* = 0.0761. Drd2-KO versus Drd2-KO + risperidone. Two-way ANOVA, Fisher’s LSD post-hoc test, risperidone, *F*(1,20) = 0.6023, *P* = 0.4468; social target, *F*(1,20) = 12.59, *P* = 0.0020; risperidone × social target, *F*(1,20) = 3.500, *P* = 0.0761. Social preference (**e**). WT versus Drd2-KO. Two-way ANOVA, Fisher’s LSD post-hoc test. Genotype, *F*(1,22) = 6.194, *P* = 0.0209; social target, *F*(1,22) = 10.82, *P* = 0.0033; genotype × social target, *F*(1,22) = 8.389, *P* = 0.0084. Drd2-KO versus Drd2-KO + LpEV. Two-way ANOVA, Fisher’s LSD post-hoc test, LpEV, *F*(1,20) = 3.568, *P* = 0.0735; social target, *F*(1,20) = 3.568, *P* = 0.0735; LpEV × social target, *F*(1,20) = 3.762, *P* = 0.0667. Drd2-KO versus Drd2-KO + risperidone. Two-way ANOVA, Fisher’s LSD post-hoc test, risperidone, *F*(1,20) = 5.822, *P* = 0.0256; social target, *F*(1,20) = 8.186, *P* = 0.0097; risperidone × social target, *F*(1,20) = 6.525, *P* = 0.0189. Female (**f** and **g**). Sociability (**f**). WT versus Drd2-KO. Two-way ANOVA, Fisher’s LSD post-hoc test. Genotype, *F*(1,20) = 21.04, *P* = 0.0002; social target, *F*(1,20) = 89.47, *P* < 0.0001; genotype × social target, *F*(1,20) = 31.75, *P* < 0.0001. Drd2-KO versus Drd2-KO + LpEV. Two-way ANOVA, Fisher’s LSD post-hoc test, LpEV, *F*(1,18) = 1.242, *P* = 0.2798; social target, *F*(1,18) = 14.92, *P* = 0.0011; LpEV × social target, *F*(1,18) = 1.562, *P* = 0.2274. Drd2-KO versus Drd2-KO + risperidone. Two-way ANOVA, Fisher’s LSD post-hoc test, risperidone, *F*(1,20) = 13.58, *P* = 0.0015; social target, *F*(1,20) = 2.720, *P* = 0.1147; risperidone × social target, *F*(1,20) = 0.7831, *P* = 0.3867. Social preference (**g**). WT versus Drd2-KO. Two-way ANOVA, Fisher’s LSD post-hoc test. Genotype, *F*(1,20) = 3.117, *P* = 0.0928; social target, *F*(1,20) = 1.650, *P* = 0.2136; genotype × social target, *F*(1,20) = 5.902, *P* = 0.0247. Drd2-KO versus Drd2-KO + LpEV. Two-way ANOVA, Fisher’s LSD post-hoc test, LpEV, *F*(1,18) = 1.396, *P* = 0.2527; social target, *F*(1,18) = 3.254, *P* = 0.0880; LpEV × social target, *F*(1,18) = 12.57, *P* = 0.0023. Drd2-KO versus Drd2-KO + risperidone. Two-way ANOVA, Fisher’s LSD post-hoc test, risperidone, *F*(1,20) = 5.482, *P* = 0.0297; social target, *F*(1,20) = 0.02442, *P* = 0.8774; risperidone × social target, *F*(1,20) = 1.651, *P* = 0.2135. **h**–**m**, Repetitive behaviors. Time spent by male (**h**–**j**) and female (**k**–**m**) Drd2-KO mice on self-directed grooming (**h** and **k**), rearing (**i** and **l**), and digging (**j** and **m**). Male: *n* = 6 animals per group. Female: WT, 7 animals; Drd2-KO, 5 animals; Drd2-KO + LpEV, 5 animals; Drd2-KO + risperidone, 7 animals. Male (**h**–**j**): WT versus Drd2-KO versus Drd2-KO + LpEV versus Drd2-KO + risperidone. One-way ANOVA, Tukey’s post-hoc test. Grooming, *F*(3,20) = 7.421, *P* = 0.0016; rearing, *F*(3,20) = 4.392, *P* = 0.0158; and digging, *F*(3,20) = 0.3340, *P* = 0.8009. Female (**k**–**m**): WT versus Drd2-KO versus Drd2-KO + LpEV versus Drd2-KO + risperidone. One-way ANOVA, Tukey’s post-hoc test. Grooming, *F*(3,20) = 7.666, *P* = 0.0013; rearing, *F*(3,20) = 5.292, *P* = 0.0075; and digging, *F*(3,20) = 1.212, *P* = 0.3311.

To assess the therapeutic potential of LpEV in autistic-like behaviors in Drd2-KO mice, LpEV was orally administered at a dose of 2.27 mg/kg/day (equivalent to 1.753 × 10^12^ particles/kg/day, or 50 μg per 22 g body weight per day) for 4 weeks (Fig. 2a). Compared with untreated Drd2-KO mice, LpEV-treated Drd2-KO mice showed significantly more interaction with a social target in the social behavior test in both males and females (Fig. 2d, e). Drd2-KO mice treated with LpEV also displayed a normal preference for the novel stranger over the familiar one (Fig. 2f, g). Furthermore, Drd2-KO mice treated with LpEV displayed reduced self-grooming compared with untreated Drd2-KO mice in both sexes. However, LpEV did not affect rearing or digging behaviors in Drd2-KO mice (Fig. 2h–m).

Risperidone was used as a reference molecule for evaluating the effectiveness of LpEV. Risperidone treatment in Drd2-KO mice improved social interaction and social novelty preference in males, but not females (Fig. 2a–g). However, risperidone-treated Drd2-KO males exhibited abnormally suppressed locomotion and locomotion speed (Fig. 2b, c). Risperidone treatment improved increased self-grooming, but it suppressed rearing in both sexes (Fig. 2h–m).

We further investigated the effects of LpEV dose. A lower dose (15 μg per 22 g body weight per day) significantly improved both social interaction with a social target and social novelty preference in Drd2-KO mice, but it did not affect self-grooming. The lowest dose (5 μg per 22 g body weight per day) showed no significant effects on either social behaviors or self-grooming (Supplementary Fig. 2).

### LpEV treatment reversed altered gene expression in the brain of Drd2-KO mice

The behavioral improvement by LpEV treatment suggests LpEV’s potential to rectify dysfunctional signaling pathways in Drd2-KO mice. To investigate the underlying molecular mechanisms, we performed RNA sequencing on key brain regions (dStr, NAc and dHP) implicated in ASD core symptoms. We used two complementary data analysis approaches. The first focused on identifying genes whose expression in Drd2-KO mice was ‘reversed’ by LpEV treatment. The second approach investigated genes that were ‘changed’ by LpEV in these mice.

**Fig. 3:**
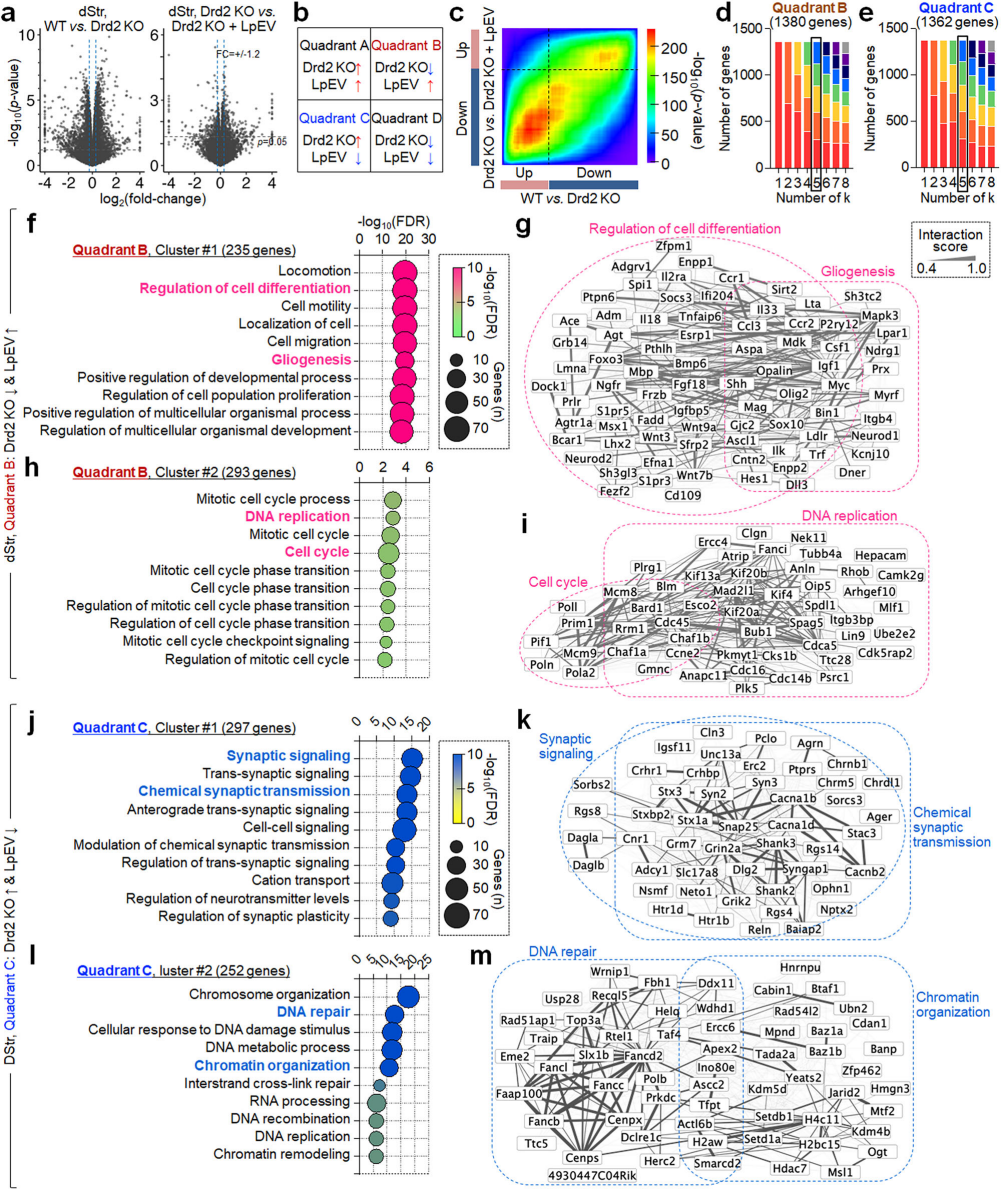
LpEV treatment reversed the altered expression of genes regulating, DNA repair, cell cycle process and cell differentiation in the dStr of Drd2-KO mice. **a**–**c**, Volcano plots of dStr RNA sequencing datasets (**a**). RRHO map depicting possible gene expression patterns following treatment with LpEV in Drd2-KO mice (**b**). A RRHO map with gene expression in the dStr of Drd2-KO mice (WT versus KO) and gene expression after LpEV treatment in Drd2-KO mice (KO versus KO +LpEV) (**c**). RNA seq data were ranked on the basis of signed log_10_-transformed *t*-test *P* values between the comparison groups. Genes exclusively located within quadrants B and C were selected for further analysis. **d**,**e**, Serial *k*-means clustering and GO enrichment analysis of genes in quadrant B (1380 genes; **d**) and quadrant C (1362 genes; **e**) grouped into multiple functional clusters with distinct BPs. We selected *k* = 5 based on stable enrichment of relevant BP terms within clusters. Further details are provided in the ‘Materials and methods’ section. **f**–**i**, Two functional clusters of genes in quadrant B. Cluster 1 (235 genes) harbored genes involved in ‘regulation of cell differentiation and gliogenesis’ (**f**). Cluster 2 (293 genes) contained genes regulating ‘DNA replication and cell cycle process’ (**h**). The interaction networks of genes within the selected BPs (red; **g** and **i**). Clusters 3, 4 and 5 in quadrant B are provided in Supplementary Fig. 3. **j**–**m**, Two functional clusters of genes in quadrant C. Cluster 1 (297 genes) contained genes regulating ‘synapse signaling and chemical synapse transmission’ (**j**). Cluster 2 (252 genes) contained genes involved in ‘DNA repair and chromatin organization’ (**l**). The interaction networks of genes within the selected BPs (blue; **k** and **m**). Clusters 3, 4 and 5 in quadrant C are provided in Supplementary Fig. 4.

In our first approach, we began by analyzing the dStr RNA sequencing data using the rank–rank hypergeometric overlap (RRHO) method^27^. This analysis identified 1380 genes downregulated and 1362 genes upregulated in Drd2-KO mice, with their altered expression reversed by LpEV. Subsequent serial *k*-means clustering followed by GO enrichment analysis revealed five clusters, each with specific biological functions, within the 1380 downregulated dStr genes (Fig. 3a–e). Cluster 1 (235 genes) contained genes involved in ‘regulation of cell differentiation and gliogenesis’, which included *IL18*, *Bmp6*, *Ngfr*, *Tnf18*, *Wnt3*, *IL33*, *Shh,* *Igf1*, *Csf1*, *Olig2*, *Sirt2*, *Myc* and *NeuroD1* (Fig. 3e, f); cluster 2 (293 genes) encompassed genes for ‘DNA replication and cell cycle process’, which included *Kif4*, *Kif20a*, *Kif20b*, *Mad2l1*, *Mcm8*, *Cdca5*, *Cdc45*, *Bard1* and *Chaf1b* (Fig. 3g, h); cluster 3 (308 genes) harbored genes regulating ‘microtubule-based process’; cluster 4 (237 genes) contained genes for ‘neuron projection development and differentiation’; and cluster 5 (289 genes) enriched genes regulating ‘membrane lipid (sphingolipid/galactolipid/glycosylceramide) biosynthetic process’ (Supplementary Fig. 3).

We then analyzed the 1362 upregulated dStr genes in Drd2-KO mice. Serial *k*-means clustering followed by GO enrichment revealed five functional clusters within 1362 upregulated dStr genes. Cluster 1 (297 genes) contained genes regulating ‘synaptic signaling and synaptic transmission’, which included *Shank2*, *Shank3*, *Syn2*, *Syn3*, *Snap25*, *Stx1a*, *Stx3*, *Grin2a* (*NMDA 2A*), *Grik2* (kainate receptor 2), *Grm7* (*mGluR7*), *Cacna1b* and *Cacnb2* (Fig. 3e, f); cluster 2 (252 genes) harbored genes regulating ‘DNA repair and chromatin organization’, which included *Setd1a*, *Setdb1*, *Kdm4a*, *Kdm4b*, *Hdac7*, *Top3a*, *Faap100*, *Fancb*, *Fancd2*, *Fancd2*, *Cenps* and *Cenpx* (Fig. 3g, h); cluster 3 (285 genes) harbored genes regulating ‘RNA processing’; cluster 4 (237 genes) contained genes regulating ‘phosphorylation and response to cytokine’; and cluster 5 (309 genes) encompassed genes ‘microtubule-based process’ (Supplementary Fig. 4).

**Fig. 4:**
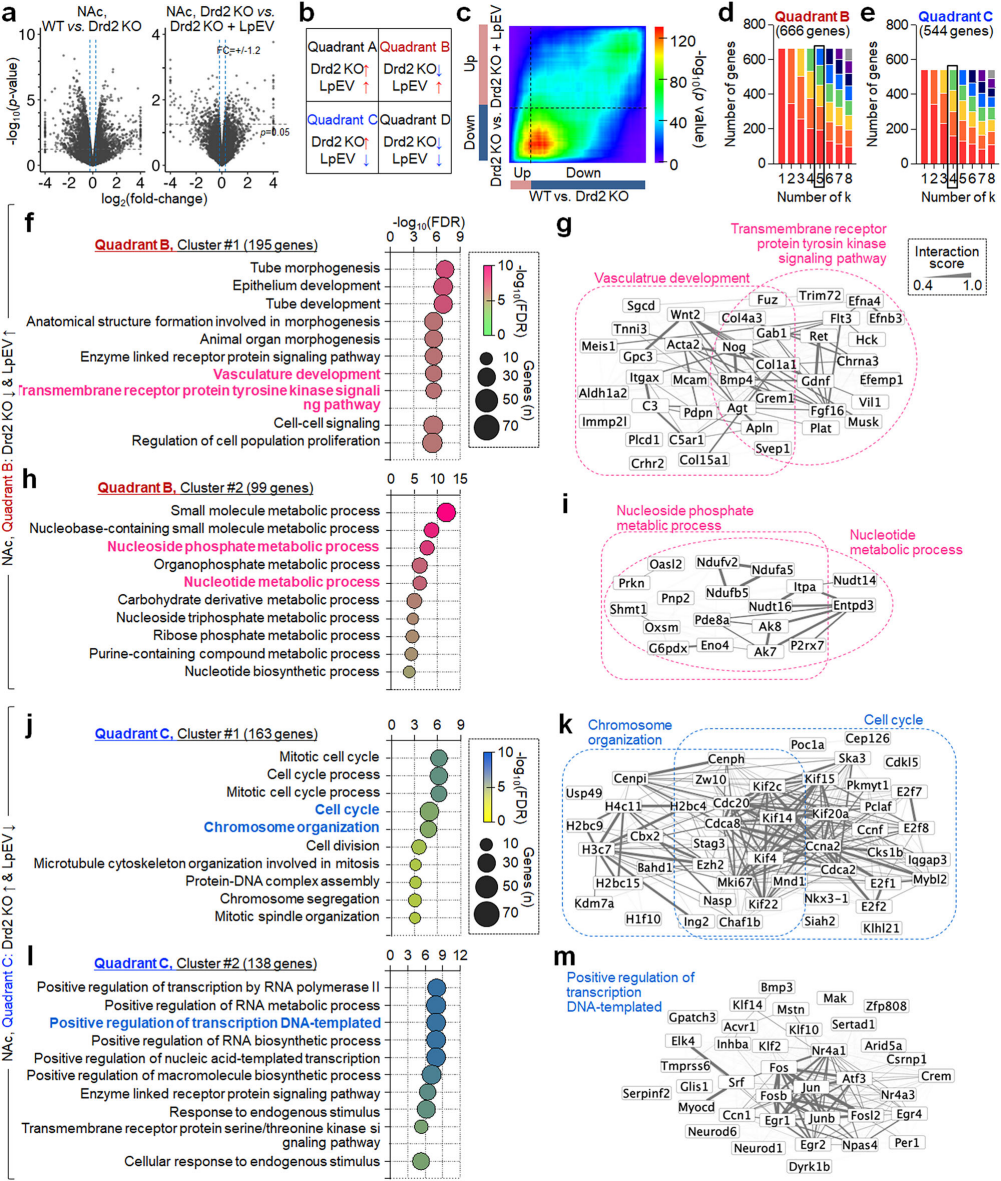
LpEV treatment reversed the altered expression of genes for vascular development and nucleotide metabolic process in the NAc of Drd2-KO mice. **a**–**e**, Volcano plots of NAc RNA seq datasets (**a**). RRHO map depicting possible gene expression patterns changed by LpEV in Drd2-KO mice (**b**). A RRHO map with gene expression in the NAc of Drd2-KO mice (WT versus KO) and gene expression changed by LpEV in Drd2-KO mice (KO versus KO + LpEV) (**c**). Genes exclusively located within quadrants B and C were selected for further analysis. **d**,**e**, Serial *k*-means clustering and GO enrichment analysis of genes in quadrant B (666 genes) (**d**) and quadrant C (544 genes) (**e**) grouped into functional clusters with distinct BPs. We selected *k* = 5. **f**–**i**, Two functional clusters of genes in quadrant B. Cluster 1 (195 genes) contained genes involved in ‘vascular development and tyrosine kinase signaling’ (**f**). Cluster 2 (99 genes) contained genes regulating ‘nucleotide metabolic process’ (**h**). The interaction networks of genes within the selected BPs (red; **g** and **i**). Clusters 3, 4 and 5 in quadrant B are provided in Supplementary Fig. 5a–f. **j**–**m**, Two functional clusters of genes in quadrant C. Cluster 1 (163 genes) harbored genes involved in ‘cell cycle and chromosome organization’ (**j**). Cluster 2 (138 genes) contained genes for ‘positive regulation of transcription, DNA-templated’ (**l**). The interaction networks of genes within the selected BPs are in blue (**k** and **m**). Clusters 3 and 4 in quadrant C are provided in Supplementary Fig. 5g–i.

RRHO analysis of NAc RNA sequencing data identified 666 downregulated and 544 upregulated genes in Drd2-KO mice, with their altered expression reversed by LpEV. We analyzed the 666 downregulated NAc genes. Subsequent serial *k*-means clustering followed by GO enrichment revealed five functional clusters within the 666 NAc genes (Fig. 4a–e). Cluster 1 (195 genes) contained genes regulating ‘vasculature development and receptor tyrosine kinase signaling pathway’, which included *Wnt2*, *Nog*, *Bmp4*, *Agt*, *Fgf16*, *Gdnf* and *Ret* (Fig. 4f, g); cluster 2 (99 genes) contained genes involved in ‘nucleoside phosphate and nucleotide metabolic process’, which included *Ndufa5*, *Ndufb5*, *Nudt16*, *Ak8*, *Itpa* and *Entpd3* (Fig. 4h, i); cluster 3 (111 genes) contained genes for ‘plasma membrane bounded cell projection assembly’; cluster 4 (125 genes) contained genes regulating ‘transmembrane transport’; and cluster 5 (136 genes) contained genes involved in ‘immune response’ (Supplementary Fig. 5).

We then focused on the 544 upregulated NAc genes in Drd2-KO mice, with reversed expression by LpEV. Serial *k*-means clustering followed by GO enrichment revealed four functional clusters within the 544 NAc genes. Cluster 1 (163 genes) contained genes regulating ‘cell cycle process and chromosome organization‘, which included *H3c7*, *H4c11*, *Cdca2*, *Cdca8*, *Cdc20*, *Kif2c*, *Kif4*, *Kif14*, *Kif20a*, *Ccna2* and *Myb12* (Fig. 4j, k); cluster 2 (138 genes) contained genes involved in ‘positive regulation of transcription, DNA-templated’, which included *Egr1*, *Egr2*, *Fos*, *Fosb*, *Jun*, *Atf3* and *Nr4a3* (Fig. 4l, m); cluster 3 (125 genes) contained genes involved in ‘synaptic transmission – glutamatergic and dopamine’ and ‘secretion’; and cluster 4 (118 genes) contained genes with no enrichment (Supplementary Fig. 5).

**Fig. 5:**
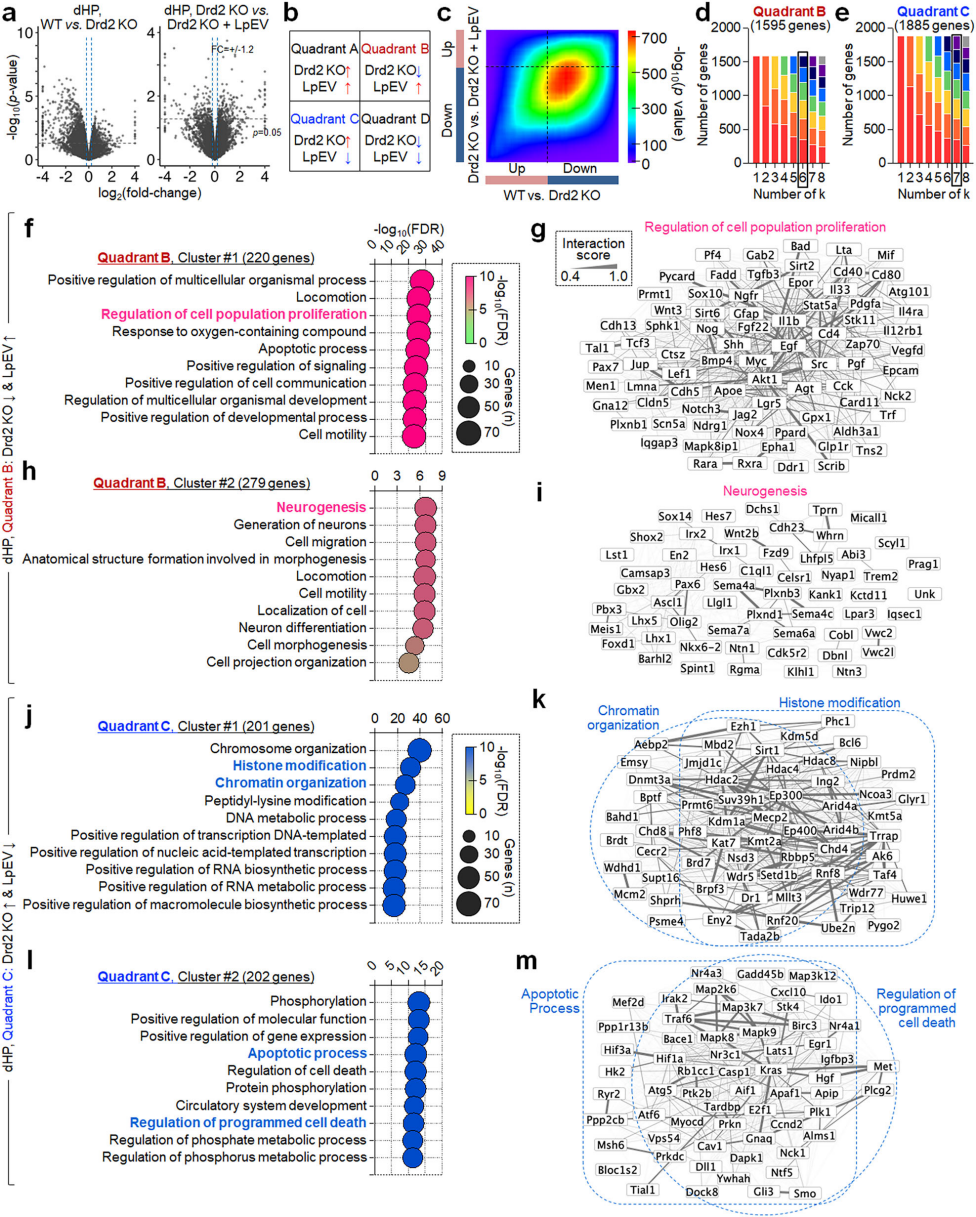
LpEV treatment reversed the altered expression of genes for cell proliferation and neurogenesis in the dHP of Drd2-KO mice. **a**–**e**, Volcano plots of dHP RNA sequencing datasets (**a**). RRHO map depicting possible gene expression patterns (**b**). A RRHO map with gene expression in the dHP of Drd2-KO mice (WT versus KO) and gene expression changed by LpEV in Drd2-KO mice (KO versus KO + LpEV) (**c**). The top 60% and 40% of ranked genes were selected from quadrants B and C, respectively, for further analysis. **d**,**e**, Serial *k*-means clustering with GO enrichment analysis revealed six and seven functional clusters (*k* = 6 and 7) with distinct BPs in quadrants B (1595 genes) (**d**) and C (1885 genes) (**e**), respectively. **f**–**i**, Two functional clusters of genes in quadrant B. Cluster 1 (220 genes) contained genes involved in ‘cell proliferation’ (**f**). Cluster 2 (279 genes) contained genes regulating ‘neurogenesis’ (**h**). The interaction networks of genes within the selected BPs are in red (**g** and **i**). Clusters 3, 4, 5 and 6 in quadrant B are provided in Supplementary Fig. 6. **j**–**m**, Two functional clusters of genes in quadrant C. Cluster 1 (201 genes) harbored genes involved in ‘histone modification and chromatin organization’ (**j**). Cluster 2 (202 genes) contained genes regulating ‘programmed cell death’ (**l**). The interaction networks of genes within the selected BPs (blue; **k** and **m**). Clusters 3, 4 and 5 in quadrant C are provided in Supplementary Fig. 7.

RRHO analysis of dHP RNA sequencing data identified 1595 downregulated and 1887 upregulated genes in Drd2-KO mice, with their expression reversed by LpEV. We first analyzed the 1595 dHP genes. Serial *k*-means clustering followed by GO enrichment revealed six functional clusters within the 1595 dHP genes (Fig. 5a–e). Cluster 1 (220 genes) contained genes for ‘regulation of cell proliferation’, which included *Bmp4*, *Shh*, *Ngfr*, *Akt1*, *Egf*, *IL1b*, *IL33*, *Notch3*, *Pdgfa*, *Stat5a*, *Sirt2*, *Sirt6* and *Myc* (Fig. 5f, g); cluster 2 (279 genes) contained genes regulating ‘neurogenesis’, which included *Wnt2b*, *Fzd9*, *C1ql1*, *Sema4a*, *Sema4c*, *Plxnb3*, *Oligo2*, *Ascl1*, *Pax6* and *Trem2* (Fig. 5h, i); cluster 3 (348 genes) contained genes regulating ‘transmembrane transport’; cluster 4 (175 genes) contained genes involved in ‘peptide biosynthetic process’ and ‘amide biosynthetic process’; cluster 5 (309 genes) contained genes regulating ‘cellular respiration’ and ‘oxidative phosphorylation’; and cluster 6 (264 genes) contained genes involved in ‘protein ubiquitination’ (Supplementary Fig. 6).

We then analyzed the 1885 dHP genes in Drd2-KO mice, with reversed expression by LpEV. Serial *k*-means clustering followed by GO enrichment revealed seven functional clusters within the 1885 dHP genes. Cluster 1 (201 genes) contained genes regulating ‘histone modification’ and ‘chromosome organization’, which included *Mecp2*, *Sirt1*, *Hdac2*, *Hdac4*, *Kdm1a*, *Jmjd1* (*Kdm3a*), *Suv39h1*, *Setdb1*, *Kmt2a*, *Ep300* and *Dnmt3a* (Fig. 5j, k); cluster 2 (202 genes) contained genes involved in ‘regulation of programmed cell death’ and ‘apoptotic process’, which included *Hif1a*, *Traf6*, *Map3k7*, *Mapk8*, *Casp1*, *Apaf1*, *Kras*, *Met* and *Nr4a3* (Fig. 5l, m); cluster 3 (243 genes) contained genes regulating ‘axon development’; cluster 4 (310 genes) contained genes involved in ‘intracellular transport’ and ‘vesicle-mediated transport’; cluster 5 (284 genes) contained genes regulating ‘cell cycle’; cluster 6 (353 genes) contained genes regulating ‘ribosome biogenesis’ and ‘RNA processing’; and cluster 7 (292 genes) contained genes involved in ‘DNA repair’ and ‘cellular response to DNA damage stimulus’ (Supplementary Fig. 7).

### Enrichment of molecular targets mediating LpEV effects within functional groups of genes

**Fig. 6:**
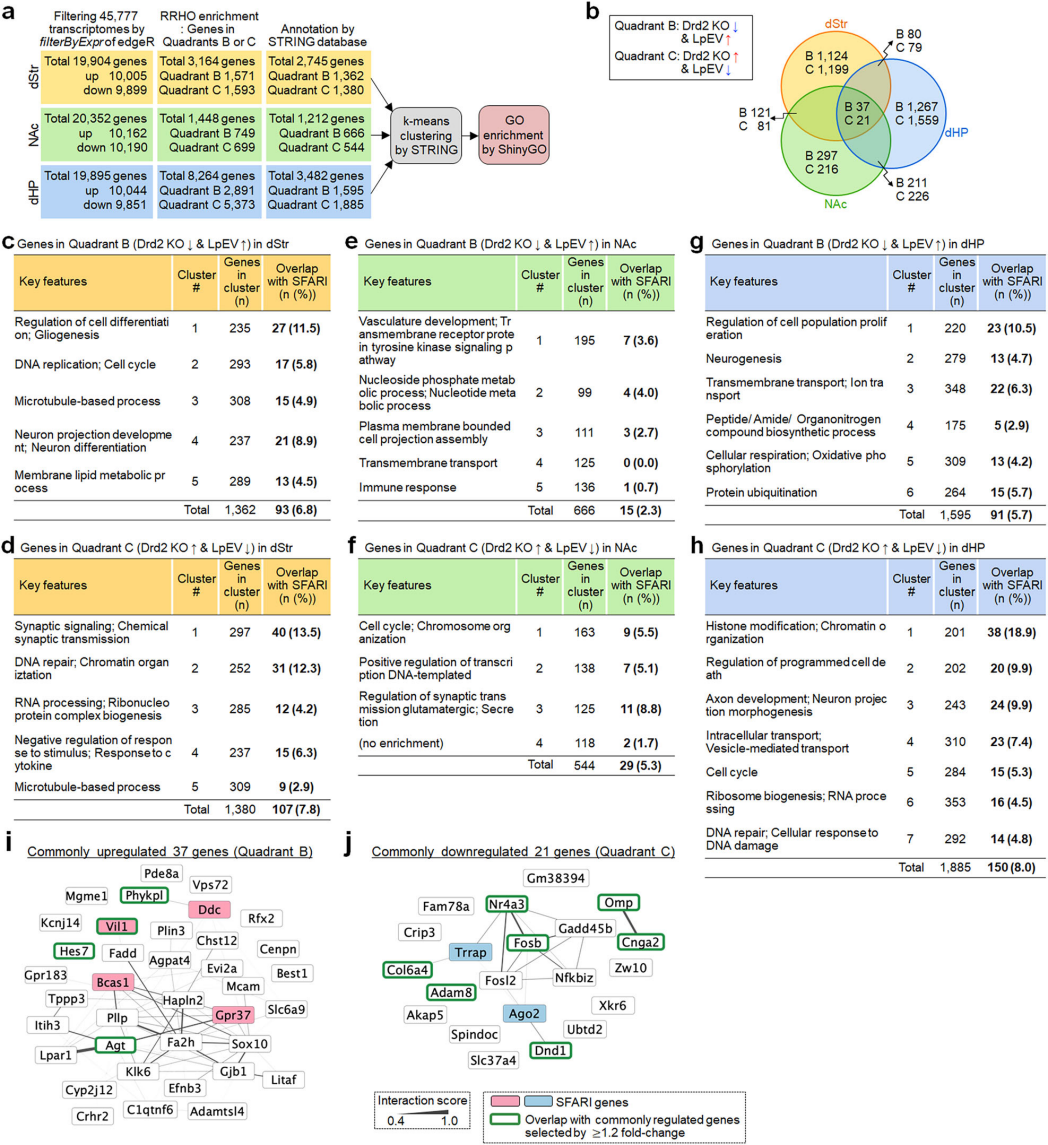
A summary of enrichment of genes whose expression was reversed by LpEV in Drd2-KO mice. **a**, **b**, Summary of selecting genes whose expression was reversed by LpEV in Drd2-KO mice, and following GO enrichment of selected genes (**a**). Genes in quadrants B and C in the RRHO map were selected for further analyses. Venn diagrams summarizing the number of genes identified in the dStr, NAc and dHP, and those overlapped between regions (**b**). **c**–**h**, Functional clusters of genes in a quadrant B in the dStr (**c**), NAc (**e**), and dHP (**g**) and a quadrant C in the dStr (**d**), NAc (**f**), and dHP (**h**). Key features in each cluster, the number of genes included and those (%) listed in the SAFRI database are summarized. **i**, **j**, The interaction networks of genes within the selected group. Genes overlapped among the dStr, NAc and dHP genes: genes (37 genes) commonly upregulated in Drd2-KO mice, with their altered expression reversed by LpEV (**i**), and those (21 genes) commonly downregulated in Drd2-KO mice, with their altered expression reversed by LpEV (**j**). Genes overlapped with SAFRI genes are marked with color shading, and those reidentified by a complementary approach (summarized in Fig. 7) are marked with green boxes.

We next explored the functional relevance of the identified genes by investigating the enrichment of SAFRI genes within specific functional groups in each brain region (Fig. 6a, b). Among the 2742 dStr genes, 200 (7.3%) overlapped with SAFRI genes. Notably, specific functional groups enriched with SAFRI genes included those for ‘regulation of cell differentiation and gliogenesis’ (11.5%), ‘neuron differentiation’ (8.9%), ‘synaptic transmission’ (13.5%) and ‘DNA repair and chromatin organization’ (12.3%). This high enrichment of SAFRI genes suggests a functional significance of these dStr gene groups in ASD (Fig. 6c, d).

Analysis of the 1,210 NAc genes revealed 44 (3.6%) SAFRI genes. Specific functional groups enriched with SAFRI genes included those for ‘cell cycle, chromosome organization’ (5.5%) and ‘regulation of synaptic transmission’ (8.8%). This suggests an importance of these identified gene groups in ASD (Fig. 6e, f).

Notably, the 3,480 dHP genes harbored 251 genes (7.2%) listed in the SFARI database. Specific functional groups encompassing genes for ‘regulation of cell proliferation’ (10.5%), ‘histone modification and chromatin organization’ (18.9%), ‘regulation of programmed cell death’ (9.9%) and ‘axon development’ (9.9%) exhibited the highest enrichment for SAFRI genes. This suggests a strong association between these dHP gene groups and ASD (Fig. 6g, h).

We further explored the shared gene expression signatures across three brain regions (Fig. 6a, b). LpEV treatment reversed the expression of 260 commonly changed genes in the dStr and NAc (representing 9.5% of dStr genes and 21.5% of NAc genes), 217 commonly affected genes in the dStr and dHP (7.9% of dStr and 6.2% of dHP genes) and 495 commonly altered genes in the NAc and dHP (40.8% of NAc and 14.2% of dHP genes) in Drd2-KO mice. Remarkably, LpEV treatment reversed the expression of 58 dysregulated genes (37 downregulated, 21 upregulated) across all three brain regions (dStr, NAc and dHP). Notably, six of these genes (*Vil*, *Ddc*, *Bcas1*, *Gpr37*, *Trrap* and *Ago2*), representing 10.3%, were in the SFARI database (Fig. 6i, j).

In summary, by analyzing shared gene expression signatures across brain regions (dStr, NAc and dHP) and specific functional groups with high prevalence of SAFRI genes, we identified various potential molecular targets that may mediate LpEV’s effects and regulate the neuronal function associated with ASD.

### Complementary strategy to identify LpEV-regulated genes in Drd2-KO mice, encompassing both genes with reversed and nonreversed expression

**Fig. 7:**
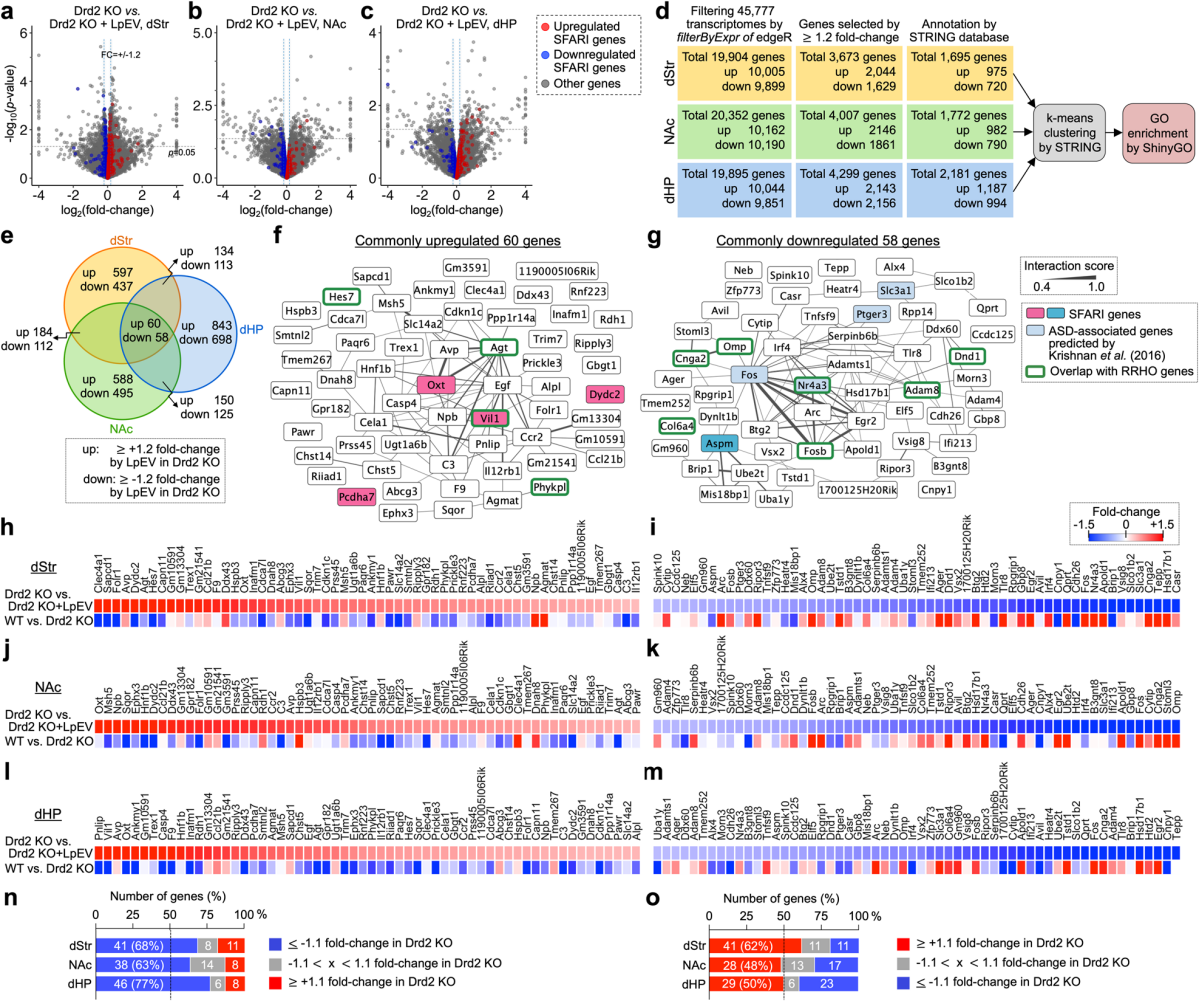
Complementary approach to identify LpEV-regulated genes in Drd2-KO mice, considering both reversal and nonreversal effects. **a**–**e**, Volcano plots showing LpEV-regulated genes in the dStr (**a**), NAc (**b**) and dHP (**c**) of Drd2-KO mice. Only genes with a fold change greater than 1.2-fold (either upregulated or downregulated) were chosen for further analysis. Genes overlapped with SFARI genes were marked with red for upregulated, blue for downregulated and gray for others. Summary of selecting LpEV-regulated genes in the dStr, NAc and dHP of Drd2-KO mice, and following GO enrichment of selected genes (**d**). Venn diagrams showing the number of LpEV-regulated genes in each brain region and those overlapped between regions (**e**). **f**, **g**, The interaction networks of genes overlappingly expressed in the dStr, NAc and dHP. Genes (60 genes) commonly upregulated across brain regions in Drd2-KO mice (**f**) and those (58 genes) commonly downregulated across brain regions in Drd2-KO mice (**g**) are indicated. Genes overlapped with SAFRI genes are marked with red for unregulated and blue for downregulated, and those overlapped with those RRHO genes (summarized in Fig. 6) are marked with open green boxes. **h**–**o**, Heat maps showing expression levels of 60 commonly upregulated in the dStr (**h**), NAc (**j**), and dHP (**l**) and 58 commonly downregulated genes after LpEV treatment in the dStr (**i**), NAc (**k**), and dHP (**m**) of Drd2-KO mice. Note that most genes unbiasedly selected as being up- or downregulated by LpEV in each brain region showed opposite expression patterns in Drd2-KO mice. The number of genes (%) were classified as upregulated (fold change ≥1.1), unchanged or downregulated (fold change ≤−1.1) in each brain region (**n** and **o**).

To identify LpEV’s potential molecular targets, we investigated gene expression changes induced by LpEV in Drd2-KO mice as a complementary approach. RNA sequencing data were filtered to focus on genes with a substantial fold change (≥1.2-fold) to ensure to analyze genes with substantial expression changes. In the dStr, this filtering identified 975 upregulated and 720 downregulated genes (total 1695). The NAc displayed a similar pattern with 982 upregulated and 790 downregulated genes (total 1772). The dHP exhibited the most notable changes, with 1187 upregulated and 994 downregulated genes (total 2181) (Fig. 7a–e). This analysis revealed a substantial overlap of LpEV-induced genes across the brain regions (Fig. 7e). Notably, 118 genes (60 upregulated and 58 downregulated), representing 7.0%, 6.7% and 5.4% of dStr, NAc and dHP genes, respectively, were consistently altered by LpEV in all three regions. Among the upregulated genes were those encoding neuropeptides associated with social and stress-related behaviors (*Oxt*, *Avp* and *Agt*), and genes involved in the clearance of damaged cells and cellular remodeling (*C3*, *Vil1*, *Dydc2* and *Pcdha7*) (Fig. 7e, f). Conversely, the downregulated genes included immediate early genes (*Aspm*, *Fos*, *Fosb* and *Arc*), known for their role in activity-dependent plasticity, and the nuclear receptor *Nr4a3* (Fig. 7f). Interestingly, five (4.2%) of these LpEV-regulated genes (*Oxt*, *Vil1*, *Dydc2*, *Pcdha7* and *Aspm*) overlapped with SFARI genes, and another four (*Fos*, *Nr4a3*, *Ptger3* and *Slc3a1*: 3.4%) were ASD-associated genes predicted by Krishnan et al. (ref. ^28^).

Examining the expression patterns of the 118 LpEV-regulated genes in Drd2-KO mice revealed a remarkable restoration after LpEV treatment (Fig. 7h–m). Among the 60 upregulated genes by LpEV, approximately two-thirds (68% in dStr, 63% in NAc and 77% in dHP) were actually downregulated by ≥1.1-fold in Drd2-KO mice. Conversely, a smaller portion (8–11% across regions) were upregulated by ≥1.1-fold in Drd2-KO mice, and 6–14% remained unchanged (Fig. 7n). Similarly, for the 58 downregulated genes by LpEV, a larger portion (62% in dStr, 48% in NAc and 50% in dHP) were upregulated in Drd2-KO mice, a relatively smaller portion (11% in dStr, 17% in NAc and 23% in dHP) were downregulated, and 6–13% remained unchanged (Fig. 7o). These results suggest that LpEV has the therapeutic potential to restore the dysregulated gene expression in Drd2-KO mice.

### LpEV treatment improved autism-like behavior in Oxtr-KO mice

RNA sequencing data (Fig. 7) suggested that LpEV might restore oxytocin signaling, which aligns with the SAFARI gene analysis implicating the oxytocin and oxytocin receptor system in the Drd2 pathway (Fig. 1l–p). To investigate the role of oxytocin signaling in LpEV’s effects, we utilized mice heterozygous for the oxytocin receptor (Oxtr^+/−^). Oxtr^+/−^ mice were generated as described in the ‘Materials and methods’ section.

**Fig. 8:**
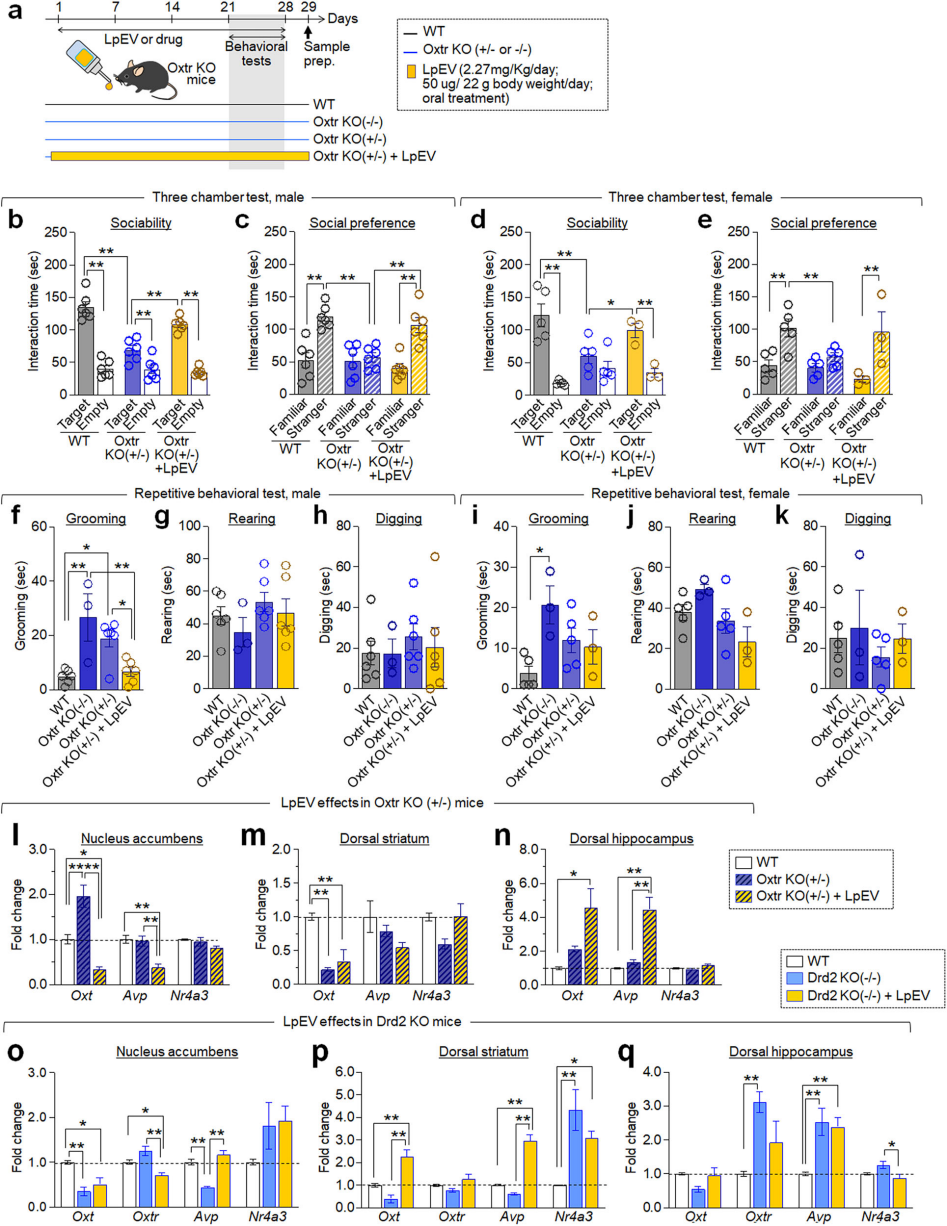
LpEV treatment improved behavioral deficits of Oxtr heterozygous mice (Oxtr^+/−^). **a**, Experimental design. Oxtr^+/−^ mice were orally administered LpEV (2.27 mg/kg/day, or 50 μg per 22 g body weight per day) for 3 weeks, and this treatment continued during behavioral testing. Behavioral tests were conducted sequentially: social interaction, social preference and repetitive tests. **b**–**e**, Social behaviors. Time spent exploring a social target compared with an empty cage (**b** and **d**) and time spent exploring a novel stranger versus a familiar one (**c** and **e**) in the three-chamber social behavior test. Data shown for male (**b** and **c**) and female (**d** and **e**) Oxtr^+/−^ mice. Male: *n* = 6 animals per group. Female: WT, 5 animals; Drd2-KO, 5 animals; Drd2-KO + LpEV, 3 animals. Male (**b** and **c**). Sociability (**b**). WT versus Oxtr^+/−^. Two-way ANOVA, Fisher’s LSD post-hoc test. Genotype, *F*(1,20) = 23.23, *P* = 0.0001; social target, *F*(1,20) = 79.82, *P* < 0.0001; genotype × social target, *F*(1,20) = 22.32, *P* = 0.0001. Oxtr^+/−^ versus Oxtr^+/−^ + LpEV. Two-way ANOVA, Fisher’s LSD post-hoc test. LpEV, *F*(1,20) = 10.36, *P* = 0.0032; social target, *F*(1,20) = 88.54, *P* < 0.0001; LpEV × social target, *F*(1,20) = 16.33, *P* = 0.0006. Social preference (**c**). WT versus Oxtr^+/−^. Two-way ANOVA, Fisher’s LSD post-hoc test. Genotype, *F*(1,20) = 12.16, *P* = 0.0023; social target, *F*(1,20) = 15.66, *P* = 0.0008; genotype × social target, *F*(1,20) = 11.05, *P* = 0.0034. Oxtr^+/−^ versus Oxtr^+/−^ + LpEV. Two-way ANOVA, Fisher’s LSD post-hoc test. LpEV, *F*(1,20) = 4.487, *P* = 0.0469; social target, *F*(1,20) = 15.52, *P* = 0.0008; LpEV × social target, *F*(1,20) = 10.94, *P* = 0.0035. Female (**d** and **e**). Sociability (**d**). WT versus Oxtr^+/−^. Two-way ANOVA, Fisher’s LSD post-hoc test. Genotype, *F*(1,16) = 2.857, *P* = 0.1104; social target, *F*(1,16) = 27.65, *P* < 0.0001; genotype × social target, *F*(1,16) = 12.85, *P* = 0.0025. Oxtr^+/−^ versus Oxtr^+/−^ + LpEV. Two-way ANOVA, Fisher’s LSD post-hoc test. LpEV, *F*(1,12) = 2.089, *P* = 0.0704; social target, *F*(1,12) = 13.79, *P* = 0.0030; LpEV × social target, *F*(1,12) = 3.944, *P* = 0.0704. Social preference (**e**). WT versus Oxtr^+/−^. Two-way ANOVA, Fisher’s LSD post-hoc test. Genotype, *F*(1,16) = 6.901, *P* = 0.0183; social target, *F*(1,16) = 16.09, *P* = 0.0010; genotype × social target, *F*(1,16) = 5.007, *P* = 0.0398. Oxtr^+/−^ versus Oxtr^+/−^ + LpEV. Two-way ANOVA, Fisher’s LSD post-hoc test. LpEV, *F*(1,12) = 0.6644, *P* = 0.4309; social target, *F*(1,12) = 11.58, *P* = 0.0052; LpEV × social target, *F*(1,12) = 4.622, *P* = 0.0527. **f**–**k**, Repetitive behaviors. Time spent by male (**f**–**h**) and female (**i**–**k**) Oxtr^+/−^ mice on grooming (**f** and **i**), rearing (**g** and **j**) and digging (**h** and **k**). Male: WT, 5 animals; Drd2-KO, 4 animals; Drd2-KO + LpEV5, 5 animals; Drd2-KO + LpEV15, 5 animals. Female: *n* = 5 animals per group. Male (**f**–**h**). WT versus Oxtr-KO (−/−) versus Oxtr-KO (+/−) versus Oxtr-KO (+/−) + LpEV. One-way ANOVA, Tukey’s post-hoc test. Grooming, *F*(3,17) = 9.423, *P* = 0.0007; rearing, *F*(3,17) = 0.8719, *P* = 0.4749; digging, *F*(3,17) = 0.2490, *P* = 0.8609. Female (**i**–**k**). WT versus Oxtr-KO (−/−) versus Oxtr-KO (+/−) versus Oxtr-KO (+/−) + LpEV. One-way ANOVA, Tukey’s post-hoc test. Grooming, *F*(3,12) = 4.502, *P* = 0.0245; rearing, *F*(3,12) = 3.064, *P* = 0.0692; digging, *F*(3,12) = 0.4702, *P* = 0.7086. **l**–**n,** RT-PCR analysis of *Oxt*, *Avp* and *Nr4a3* expression in the dStr, NAc and dHP of Oxtr^+/−^ mice after LpEV treatment. *n* = 4 animals per group; 4 repeats per group. WT versus Oxtr-KO (+/−) versus Oxtr-KO (+/−) + LpEV. One-way ANOVA, Tukey’s post-hoc test. NAc (**l**): *Oxt*, *F*(2,9) = 26.15, *P* = 0.0002; *Avp*, *F*(2,9) = 13.51, *P* = 0.0019; *Nr4a3*, *F*(2,9) = 2.348, *P* = 0.1511. dStr (**m**): *Oxt*, *F*(2,9) = 16.57, *P* = 0.0010; *Avp*, *F*(2,8) = 2.927, *P* = 0.1112; *Nr4a3*, *F*(2,9) = 3.725, *P* = 0.0663. dHP (**n**): *Oxt*, *F*(2,9) = 7.423, *P* = 0.0125; *Avp*, *F*(2,9) = 19.50, *P* = 0.0005; *Nr4a3*, *F*(2,9) = 4.206, *P* = 0.0513. **o**–**q**, RT-PCR analysis of *Oxt*, *Oxtr*, *Avp* and *Nr4a3* expression in the dStr, NAc and dHP of Drd2-KO mice after LpEV treatment. *n* = 4 animals per group; ~4–6 repeats per group. WT versus Oxtr-KO (+/−) versus Oxtr-KO (+/−) + LpEV. One-way ANOVA, Tukey’s post-hoc test. NAc (**o**): *Oxt*, *F*(2,10) = 13.19, *P* = 0.0016; *Oxtr*, *F*(2,11) = 13.69, *P* = 0.0010; *Avp*, *F*(2,9) = 33.49, *P* < 0.0001; *Nr4a3*, *F*(2,9) = 1.993, *P* = 0.1921. dStr (**p**): *Oxt*, *F*(2,13) = 20.61, *P* < 0.0001; *Oxtr*, *F*(2,11) = 3.297, *P* = 0.0755; *Avp*, *F*(2,15) = 63.59, *P* < 0.0001; *Nr4a3*, *F*(2,15) = 9.502, *P* = 0.0022. dHP (**q**): *Oxt*, *F*(2,15) = 2.908, *P* = 0.0856; *Oxtr*, *F*(2,12) = 6.850, *P* = 0.0104; *Avp*, *F*(2,15) = 8.754, *P* = 0.0030; *Nr4a3*, *F*(2,15) = 5.093, *P* = 0.0205.

Male Oxtr^+/−^ mice showed a partial impairment in social interaction in the three-chamber test, whereas female Oxtr^+/−^ exhibited severe deficits. Both male and female Oxtr^+/−^ mice displayed deficits in social novelty preference (Fig. 8a–e). Oxtr^+/−^ males also showed moderately increased grooming, while females showed a subtle increase of grooming. Their rearing and digging behaviors remained similar to wild-type mice (Fig. 8f–k). Homozygous Oxtr-KO (Oxtr^−/−^) mice displayed excessive grooming behavior in both sexes, but their rearing and digging behaviors did not differ from wild type (Fig. 8f–k). However, LpEV treatment for 3 weeks significantly improved social interaction and restored social novelty preference in both sexes in Oxtr^+/−^ mice. LpEV treatment in Oxtr^+/−^ mice also reduced excessive self-grooming in males, but not in females (Fig. 8a–k).

RT-PCR analysis revealed that both Oxtr^+/−^ mice and Drd2-KO mice displayed altered expression of ASD-associated genes, including *Oxt*, *Avp* and *Nr4a3*, in the dStr, NAc and/or dHP. By contrast, LpEV treatment in these mice changed the expression of these genes, although the specific expression patterns and LpEV-dependent changes varied between brain areas. In Oxtr^+/−^ mice, LpEV treatment counteracted the increased Oxt expression in the NAc and enhanced further its increased expression in the dHP, with no change in the Str. In Drd2-KO mice, LpEV treatment reversed its reduced expression in the dStr and dHP (Fig. 8l–q).

### LpEV treatment mitigated autism-like behaviors in Adcy5-KO mice

Adcy5 is an essential mediator for Drd2 receptor^14^, mu opioid receptors^15^ and mGluR5^12^. Furthermore, Adcy5-KO mice exhibited typical autism-like behaviors^12^. We tested whether LpEV treatment could improve autism-like behaviors in Adcy5-KO mice.

In the social behavior test, both male and female Adcy5-KO mice displayed deficits in sociability and social novelty preference. By contrast, Adcy5-KO mice treated with LpEV for 3 weeks exhibited significantly increased social interaction and social novelty preference in both sexes (Supplementary Fig. 8). Adcy5-KO mice also showed significantly increased self-grooming and increased rearing behavior compared with wild-type mice. By contrast, Adcy5-KO mice treated with LpEV showed reduced self-grooming, although their rearing was not changed by LpEV (Supplementary Fig. 8).

### LpEV treatment restored reduced Oxt expression and alleviated social behavior in Shank3-KO mice

To further investigate the therapeutic potential of LpEV, we examined its effects in Shank3-KO mice, a genetic model of ASD targeting a postsynaptic scaffolding protein in excitatory synapses, while Drd2-KO, Adcy5-KO and Oxtr-KO primarily affect GPCRs and their associated signaling pathways. Shank3-KO mice exhibited deficits in sociability and social novelty preference. Notably, LpEV treatment for 3 weeks significantly ameliorated these impairments in Shank3-KO mice. Shank3-KO mice also displayed increased grooming and reduced rearing compared with wild-type mice. Intriguingly, the LpEV treatment increased grooming in this model, while their rearing and digging remained unaffected (Supplementary Fig. 9). These results suggest that LpEV for 3 weeks improves selectively sociability behaviors in Shank3-KO mice.

We investigated the impact of LpEV on Oxt and Oxtr expression in Shank3-KO mice. Immunohistochemical analysis using anti-Oxt revealed decreased Oxt expression in the NAc, hippocampus and, to a lesser extent, the dStr of ShanK3-KO mice. By contrast, LpEV treatment led to upregulation of the reduced Oxt expression in the NAc and hippocampus. Immunohistochemical analysis using anti-Oxtr indicated that Oxtr was conversely upregulated in the NAc, and hippocampus, but not dStr, of ShanK3-KO mice, whereas LpEV treatment reversed this upregulation (Supplementary Fig. 9).

## Discussion

LpEV treatment significantly ameliorated behavioral deficits in sociability, social novelty preference and repetitive behaviors in Drd2-KO mice (Fig. 2 and Supplementary Fig. 2). We further demonstrated that LpEV treatment reversed the dysregulated expression of various groups of genes including Oxt, a neuropeptide critical for social behavior, in Drd2-KO mice (Figs. 3–7). These findings raise several interrelated issues on the therapeutic potential of LpEV and therapeutic targets of ASD.

First, LpEV treatment markedly improved autistic-like behaviors in mice with mutations in ASD-linked genes (Drd2, Oxtr, Adcy5 and Shank3) (Figs. 2 and 8 and Supplementary Figs. 2, 8 and 9). These genetic ASD models probably exhibit both accumulated deficits in early development and neural dysfunction in adulthood, similar to those seen in individuals with a typical genetic cause of ASD. Although the present study primarily focused on investigating LpEV’s therapeutic effects in Drd2-KO mice and Oxtr^+/−^ mice, it is worth noting the importance of LpEV’s therapeutic effects in Adcy5-KO mice and Shank3-KO mice. Adcy5 integrates signals from various G-protein-coupled receptors (including Drd2, mu opioid receptor and mGluR5 in the striatum) into intracellular second messengers^12,14,15^. Mu opioid receptor and mGluR5 are also associated with ASD^29,30^, underscoring the significance of LpEV’s therapeutic effects in Adcy5-KO mice. Shank3 is a postsynaptic scaffolding protein responsible for anchoring excitatory neurotransmitter receptors and other signaling molecules to the postsynaptic density^31,32^. Genetic mutations in this gene in humans cause a developmental disorder characterized by severe language problems, global developmental delay and autistic behaviors^31,32^. As demonstrated, Shank3-KO mice exhibit autistic-like core symptoms. By contrast, LpEV treatment for 3 weeks restored social behavior deficits in this model, although it enhanced grooming behavior (Supplementary Fig. 9). The precise mechanisms underlying these complex behavioral responses in Shank3-KO mice remain unclear. Considering that LpEV treatment upregulated the downregulated Oxt expression and conversely downregulated the upregulated Oxtr expression in the NAc, dStr and/or dHP of Shank3-KO mice, but these contrasting effects were not consistently observed across all regions, those complex behavioral phenotypes could result from imbalanced levels of multiple factors induced by LpEV within brain regions involved in grooming behavior. Further investigation is needed to determine whether longer-term LpEV treatment can alleviate repetitive behavioral deficits. Overall, LpEV treatment for 3 weeks effectively alleviated core ASD-like symptoms across Drd2-KO, Oxtr-KO and Adcy5-KO mice, as well as partially in Shank3-KO mice. Thus, LpEV treatment effectively alleviated core ASD-like symptoms across different genetic ASD models, suggesting its therapeutic potential for improving core ASD symptoms in a wider treatment window.

Second, we demonstrated that, while Drd2-KO disrupted the expression of numerous genes important in regulating fundamental for neural development and synaptic function, LpEV treatment reversed these changes in distinct groups of genes, including those in the SFARI database as ASD-linked genes, supporting the notion that these LpEV-responsive genes probably contribute to the observed behavioral improvements. Given that the therapeutic effects of LpEV in the genetic models of Drd2, Oxtr, Adcy5 and Shank3 genes are achieved not because of rescuing mutated gene function, it is important to understand in more detail how LpEV counteracts the accumulated neural dysfunction in the brain of these models. It is crucial to investigate whether the neural function improvements induced by LpEV in these genetic models are sustained over time or if they revert to pretreatment levels after LpEV treatment is discontinued. In addition, identifying genes that exhibit reversibility or long-term resilience to LpEV-induced neural function improvements can provide valuable insights into the underlying mechanisms of ASD. This knowledge can not only enhance the efficacy of LpEV but also inspire the development of novel therapeutic intervention for ASD.

Third, we demonstrated a substantial overlap (10–20%) between LpEV-responsive genes in specific brain regions (dStr and dHP) and genes listed in the SFARI database as ASD-linked (Figs. 6 and 7). This substantial overlap strengthens not only the LpEV’s therapeutic potential to target relevant pathways in ASD but also the importance of these regions in regulating ASD symptoms. Interestingly, the identified genes are involved in critical functions, including cell differentiation, gliogenesis, neuron development and synaptic signaling in the dStr and cell proliferation, axon development, histone modification and chromatin organization in the dHP (Fig. 6). Although the specific functions of these identified genes require further investigation, they are strong candidates as molecular targets mediating both LpEV’s therapeutic effects and ASD pathology.

We identified Oxt and its receptor Oxtr as high-confidence interactors with Drd2, through two independent approaches: screening the SFARI database of known ASD genes (Fig. 1l–p) and RNA sequencing analysis (Fig. 7). Supporting these, mice heterozygous for Oxtr (Oxtr^+/−^ mice) exhibited autistic-like behaviors. Notably, LpEV treatment in Oxtr^+/−^ mice reversed autistic-like behaviors (Fig. 8). In addition, Shank3-KO mice exhibited reduced Oxt expression in the NAc and dHP (Supplementary Fig. 9). Further analysis indicated that LpEV treatment restored the reduced Oxt expression across multiple brain regions in Oxtr^+/−^ mice, Drd2-KO mice and Shank3-KO mice, although the specific regional effects appeared more complex than this simplification. While our study has primarily focused on the NAc, dStr and dHP, it is possible that the overall brain-wide Oxt system plays a role in regulating autistic-like behaviors. Oxytocin neurons are predominantly located in the paraventricular nucleus and supraoptic nucleus of the hypothalamus and the bed nucleus of the stria terminalis and, to a lesser extent, in the BNST, NAc, hippocampus and amygdala^33,34^. By contrast, oxytocin receptors are expressed widely in the brain, including the prefrontal cortex, other cerebral areas, lateral septum, dStr, NAc, BNST, hippocampus, midbrain regions and brainstem^33,34^. Consequently, the NAc, dStr and dHP are influenced by local Oxt neurons, but also synaptic inputs from hypothalamic Oxt neurons. Considering the complexity of the Oxt system in the brain, further research is needed to understand the precise role of local Oxt neutrons in the NAc, dStr and dHP and that of hypothalamic Oxt neutrons in regulating ASD-associated behaviors and their roles in mediating LpEV’s effects.

Recently, we reported that LpEV treatment reversed glucocorticoid- and Aβ42-induced genome-wide transcriptional alterations in HT22 neuronal cells^22,23^ and also restored Aβ pathology in the brain of Tg-APP/PS1 mice^23^. As demonstrated in the present study, LpEV had the ability to normalize the altered expression of genes associated with cell proliferation, gliogenesis, neuronal development and synaptic signaling as well as nuclear factors including histone modification and chromatin organization in Drd2-KO mice (Figs. 2–6). These results suggest that LpEV possesses a broad spectrum of biological effects to counteract molecular and cellular dysfunction in neural cells. Future investigations may be needed to elucidate whether LpEV’s diverse effects are induced by its multiple bioactive components or by LpEV’s ability to activate a central pathway that triggers multiple downstream signaling events.

While the presence of radioactive-labeled LpEV in the brain after oral administration (Supplementary Fig. 1) suggests its potential to directly target brain cells, further research is needed to determine if LpEV can also exert therapeutic effects indirectly through the peripheral system. Addressing these questions will be helpful for optimizing LpEV’s therapeutic potential.

Given the oral administration of LpEV, it is plausible that LpEV could exert its effects through the mechanisms implicated in gut–brain axis communication. The gut microbiome influences brain function through various pathways. These include (1) neuroanatomical pathways, such as retrograde transport of bacterial metabolites directly through the vagus nerve innervating gut epithelial cells, and subsequent changes of neural function^35,36^; (2) immunological pathways, such as through resident immune cells in the gut lining releasing immunoactive cytokines^37^; (3) microbial-derived products, such as short-chain fatty acids, carbohydrates, bile acid metabolites, dopamine, GABA and tryptophan metabolites^38,39^; (4) neuroendocrine (gut hormone) signaling stimulated by bacterial products^40^; and (5) bacteria-derived EVs^19,22,23^.

LpEV had an average diameter of 181 ± 3.0 nm, as measured by a dynamic light scattering method. LpEV’s nanosize and liposome-like nature allow it to cross the gut barrier and penetrate various biological barriers, including the blood–brain barrier^41^. Our biodistribution data of isotope-labeled LpEV support this notion (Supplementary Fig. 1). In addition, several lines of accumulated in vitro and in vivo data support the potential of EVs to target brain cells directly and activate specific signaling pathways in the brain^22,23^. However, this study acknowledges the limitation of elucidating the precise mechanisms underlying LpEV’s effects without identifying the specific bioactive components within these vesicles. Standardization of LpEV production is crucial, as isolation techniques (for example, ultrafiltration and ultracentrifugation) and culture conditions (media, temperature, duration and aerobic/anaerobic) significantly impact EV quality. While certain biomarkers might be used for quality control, they are suitable when related to the bioactive components. In this study, LpEV was prepared using ultrafiltration and ultracentrifugation (detailed in the Supplementary Information). LpEV concentration was determined using both physical (dynamic light scattering) and biochemical (Bradford assay) methods. Furthermore, we assessed the biological activity of LpEV from different batches by examining their consistent transcriptional effects on MeCP2 and other epigenetic factors in HT22 cells. This approach may help to ensure the preparation of standardized LpEV until the bioactive components are fully elucidated.

Although there is no widely agreed-upon control for evaluating the therapeutic effects of anti-ASD drugs in animal models of ASD, this study utilized risperidone as a reference point. Risperidone is a clinically approved medication for alleviating irritability in individuals with ASD. While earlier animal model studies reported no improvements in repetitive or social behaviors, recent research suggests that risperidone can alleviate core ASD symptoms, including sociability and grooming behaviors, in animal models. It appears that long-term treatment with low doses of risperidone (0.1–0.25 mg/kg/day for 1–4 weeks) has yielded beneficial effects on autistic-like behaviors in animal models such as the valproic acid model and mu opioid receptor KO mice^29,42–45^. Our findings partially align with these recent studies, demonstrating improvements in grooming and social behaviors in male Drd2-KO mice after risperidone treatment (intraperitoneal, 0.2 mg/kg/day) over 8 days. However, risperidone treatment in female Drd2-KO mice suppressed locomotor activity and significantly reduced social exploration, indicating severe side effects at this dose. These results suggest that risperidone may have a limited utility as a consistent positive control in preclinical studies of ASD. In light of these observations, LpEV, or its yet unidentified active components, may serve as a more suitable positive control for evaluating the efficacy of potential ASD drug candidates. Further investigation into the specific bioactive components of LpEV and their mechanisms of action is crucial to fully understand its potential as a control in preclinical research.

Overall, LpEV’s ability to restore dysregulated genes in brain regions linked to ASD behaviors holds a promise for treating the core symptoms beyond symptomatic management. Further investigation is needed to understand the detailed mechanisms by which LpEV reverses cellular processes and modifies neural function, and to improve our knowledge of the active components inducing those effects.

## Supplementary information

Supplementary Information

## Data Availability

Raw data and materials will be made available on reasonable request.
