# Supplementary material for: Lactobacillus paracasei-derived extracellular vesicles reverse molecular and behavioral deficits in mouse models of autism spectrum disorder
Source: Exp Mol Med. 2025 Apr 1;57(4):788–803. doi: 10.1038/s12276-025-01429-w (PMC12045967; doi:10.1038/s12276-025-01429-w)
Supplement: Supplementary file 1 — Supplementary Information [file 12276_2025_1429_MOESM1_ESM.pdf]

SUPPLEMENTARY INFORMATION

Lactobacillus paracasei-derived extracellular vesicles reverse molecular and behavioral deficits in mouse models of autism spectrum disorder

Park, J.Y. et al.

Corresponding authors: Dr. TK Kim (spt21@knsu.ac.kr),
Dr. YK Kim (e-mail: ykkim@mdhc.kr), and
Dr. PL Han (e-mail: plhan@ewha.ac.kr)

Illustrations: Supplementary Figures 1,2,3,4,5,6,7,8,9
Supplementary Materials and Methods

Supplementary Fig. 1

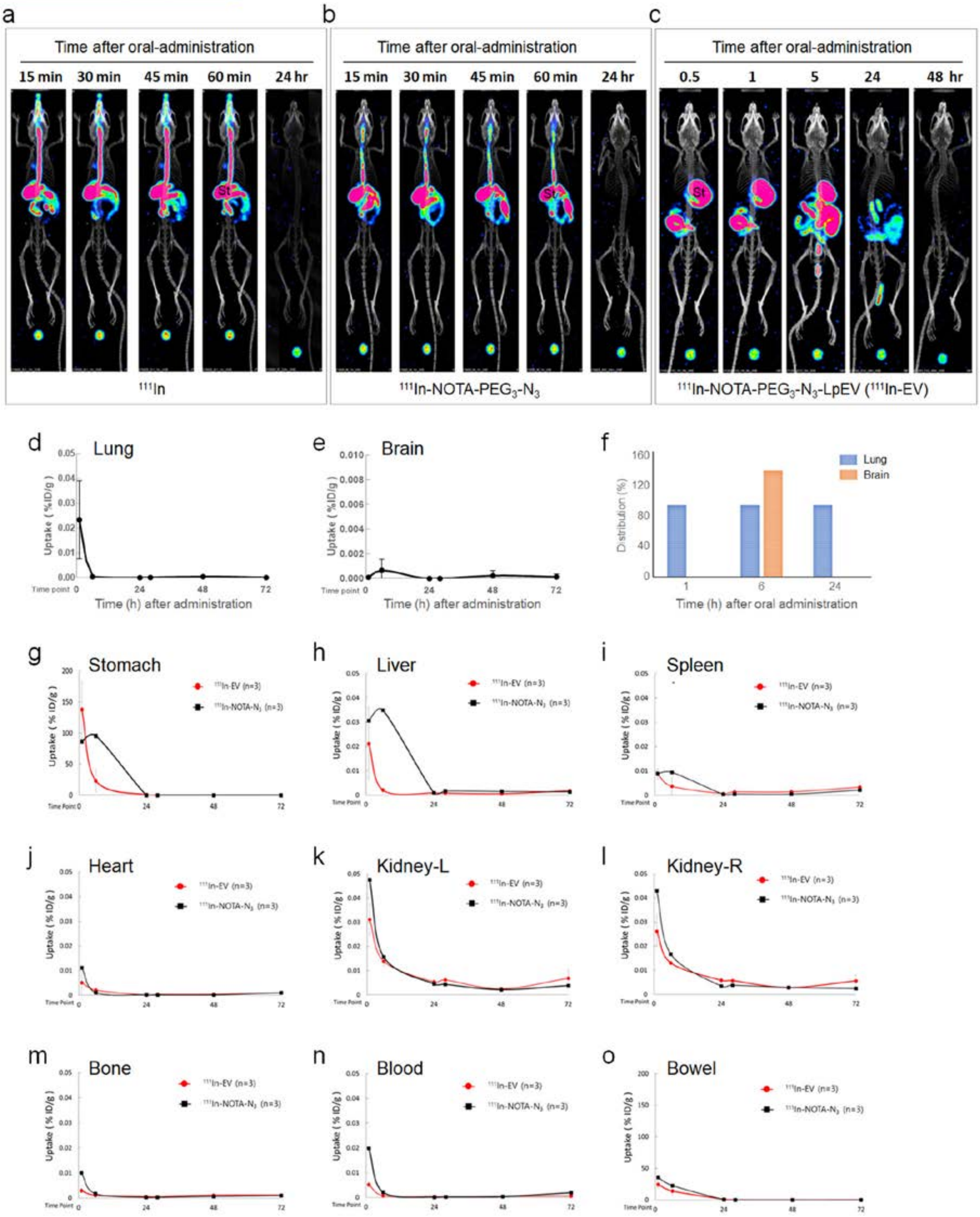

Supplementary Fig. 1. Whole body imaging the biodistribution of orally administered isotope-labeled LpEV in mice.

a-c Whole body imaging of Indium-111 (^{111}In) (**a**), ^{111}In-NOTA-PEG$_3$-N$_3$ (**b**), and ^{111}In-NOTA-PEG$_3$-N$_3$-LpEV (^{111}In-LpEV) (**c**) biodistribution at indicated time points post-oral administration. Note that orally administered ^{111}In-LpEV persisted in the

digestive system and kidneys at 24 hours post-oral administration, albeit at reduced levels, while ^{111}In and ^{111}In-NOTA-PEG_3-N_3 were cleared by this time point.

d-f Radioactivity, measured by a gamma counter, of ^{111}In-NOTA-PEG_3-N_3-LpEV (^{111}In-LpEV) in the lungs and brain (**d,e,f**) of mice at indicated time points post-oral administration.

g-o Radioactivity, measured by a gamma counter, of ^{111}In-NOTA-PEG_3-N_3 (black) and ^{111}In-NOTA-PEG_3-N_3-LpEV (red) in the stomach (**g**), liver (**h**), spleen (**i**), heart (**j**), kidney-L (**k**), kidney-R (**l**), bone (**m**), blood (**n**), and bowel (**o**) of mice at indicated time points post-oral administration.

Supplementary Fig. 2

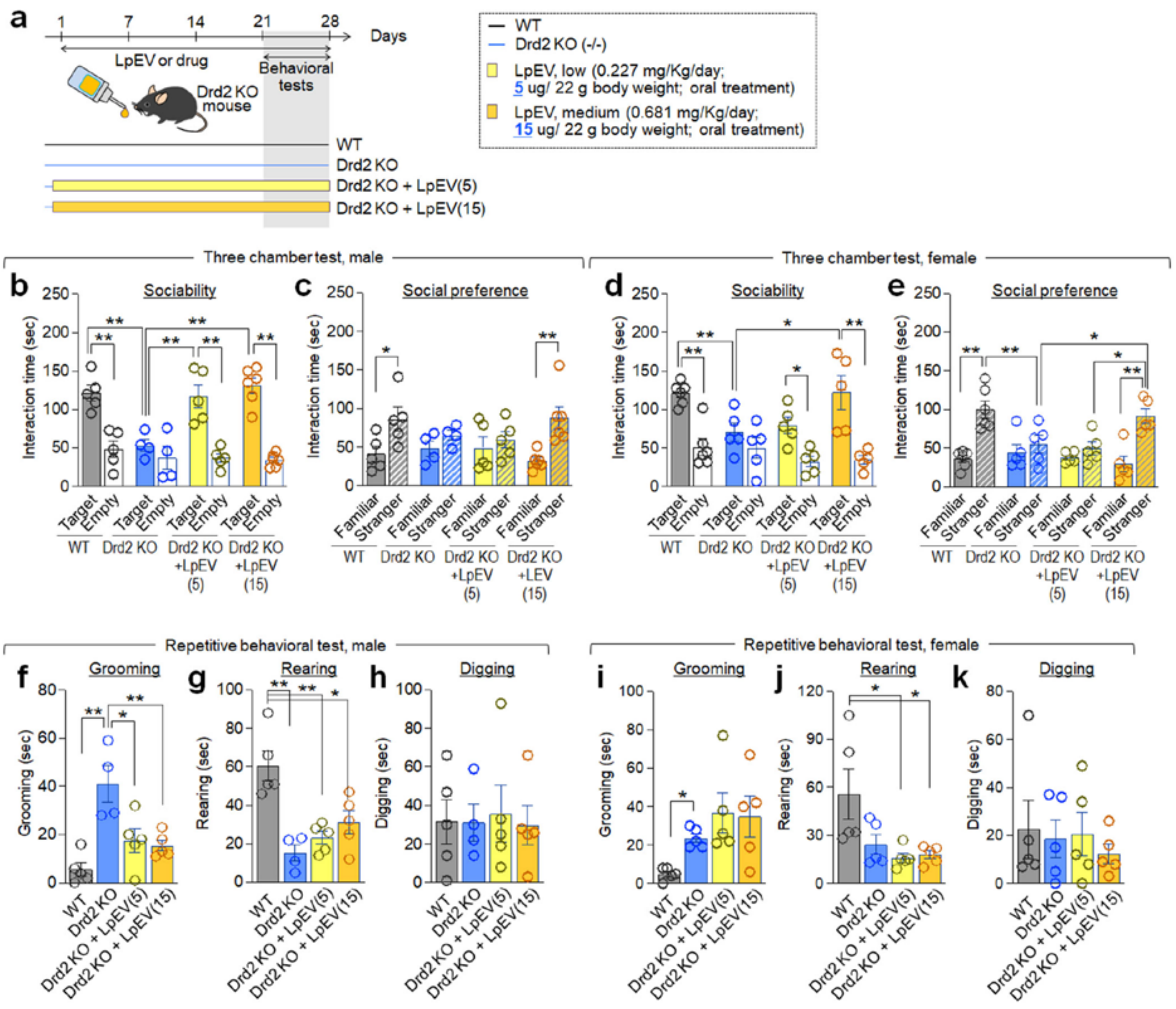

Supplementary Fig. 2. LpEV treatment at low-doses partially improved behavioral deficits of Drd2 KO mice.

a Experimental design. Drd2 KO mice were administrated LpEV orally at low dose (0.227 mg/Kg/day; 5 μg/ 22 g body weight/day) or at medium dose (0.681 mg/Kg/day; 15 μg/ 22 g body weight/day) for 3 weeks, continuing during behavioral testing. Behavioral tests were conducted in the order of social interaction, social preference, and repetitive tests. 5 and 15, denote low and medium doses.

b-e. Social behaviors. Time spent exploring a social target compared to an empty cage (**b,d**) and time spent exploring a novel stranger versus a familiar one (**c,e**) in the three-chamber sociability test. Data shown for male (**b,c**) and female (**d,e**) Drd2 KO mice. Male: WT, 5 animals; Drd2 KO, 4 animals; Drd2 KO + LpEV5, 5 animals; Drd2 KO + LpEV15, 6 animals. Female: WT, 6 animals; Drd2 KO, 5 animals; Drd2 KO + LpEV5, 5 animals; Drd2 KO + LpEV15, 5 animals. Mean +/- SEM. *, $p < 0.05$; **, $p < 0.01$ (Two-way ANOVA, Fisher's LSD post-hoc test).

f-k. Repetitive behaviors. Time spent by male (**f,g,h**) and female (**i,j,k**) Drd2 KO mice on self-directed behaviors of grooming (**f,i**), rearing (**g,j**), and digging (**h,k**). Male: WT, 5 animals; Drd2 KO, 4 animals; Drd2 KO + LpEV5, 5 animals; Drd2 KO + LpEV15, 5 animals. Female: WT, 5 animals; Drd2 KO, 5 animals; Drd2 KO + LpEV5, 5 animals; Drd2 KO + LpEV15, 5 animals. Mean +/- SEM. *, $p < 0.05$; **, $p < 0.01$ (One-way ANOVA, Tukey's post-hoc test).

Supplementary Fig. 3

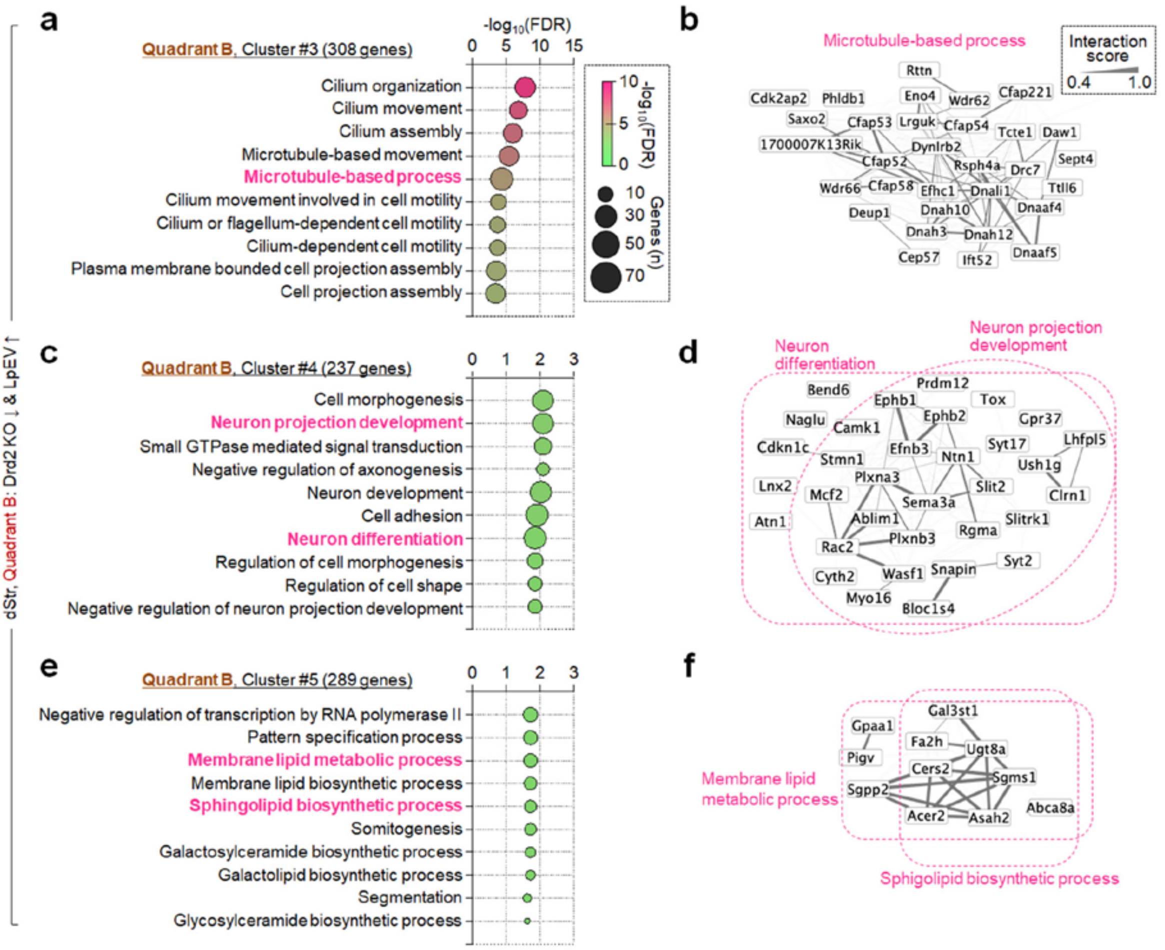

Supplementary Fig. 3. Three clusters in Quadrant B identified in the dorsal striatum of Drd2 KO mice and Drd2 KO mice treated with LpEV.

a-f Top 10 biological processes enriched in each of the remaining three clusters of genes identified in Quadrant B. Cluster #3 (308 genes) contained genes regulating microtubule-based process (**a**). The interaction network of genes covered by the selected biological process in red (**b**); Cluster #4 (237 genes) encompassed genes regulating neuron development and differentiation (**c**). The interaction network of genes within the biological process marked in red (**d**); and Cluster #5 (289 genes) enriched genes involved in membrane lipid metabolic process (**e**). The interaction network of genes within the biological process underlined in red (**f**). Clusters #1 and #2 are provided in **Fig. 3f-i**.

Supplementary Fig. 4

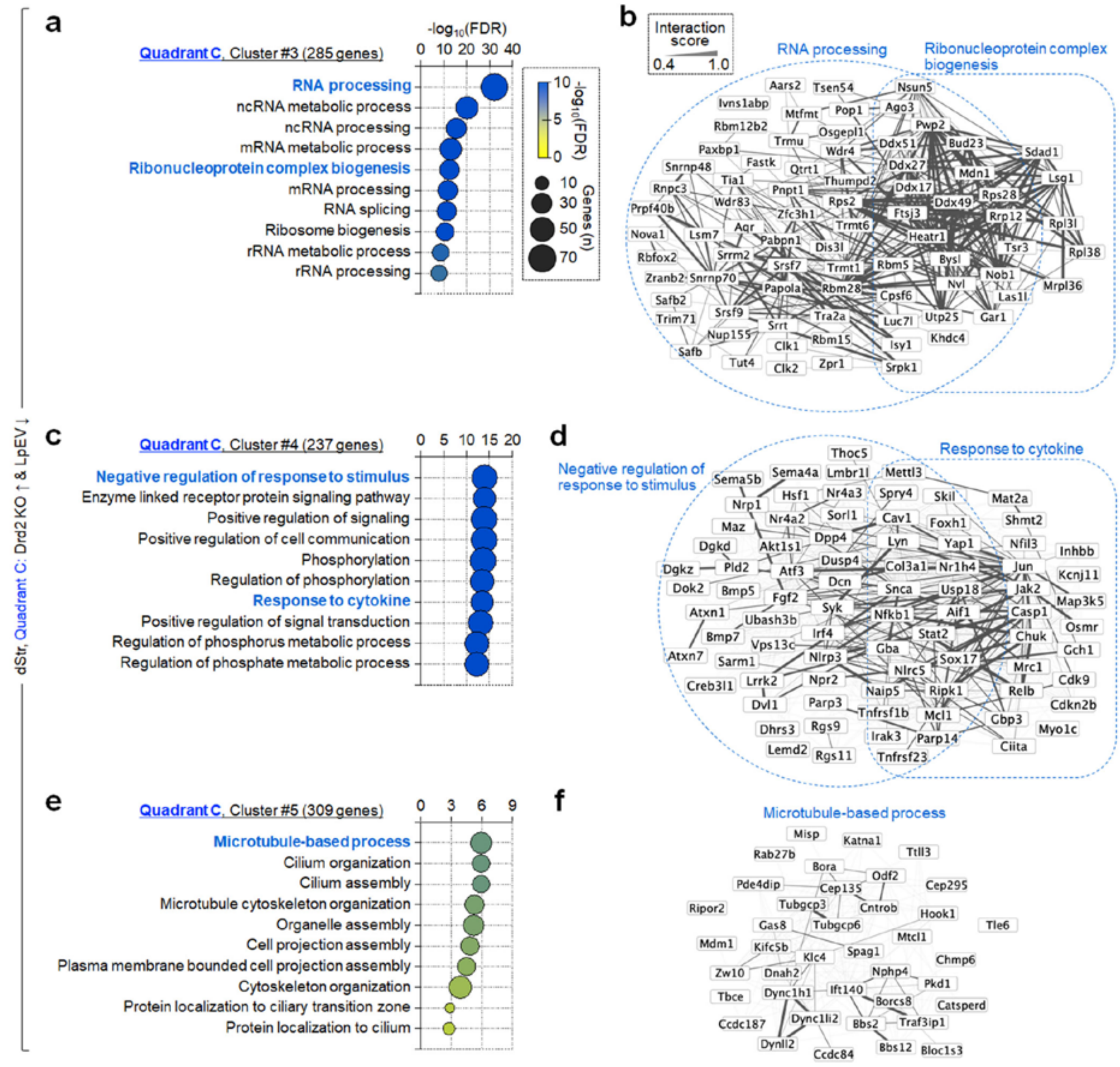

Supplementary Fig. 4. Three clusters in Quadrant C identified in the dorsal striatum of Drd2 KO mice and Drd2 KO mice treated with LpEV..

a-f Top 10 biological processes in each of the remaining three clusters of genes identified in Quadrant C. Cluster #3 (285 genes) contained genes regulating RNA processing and response to cytokine (**a**). The interaction network of genes within the biological process underlined in blue (**b**); Cluster #4 (237 genes) encompassed genes for negative regulation of response to stimulus and response to cytokine (**c**). The interaction network of genes within the selected biological process in blue (**d**); and Cluster #5 (309 genes) harbored genes regulating microtubule-based process (**e**). The interaction network of genes within the biological process in blue (**f**). Clusters #1 and #2 are presented in **Fig. 3j-m**.

Supplementary Fig. 5

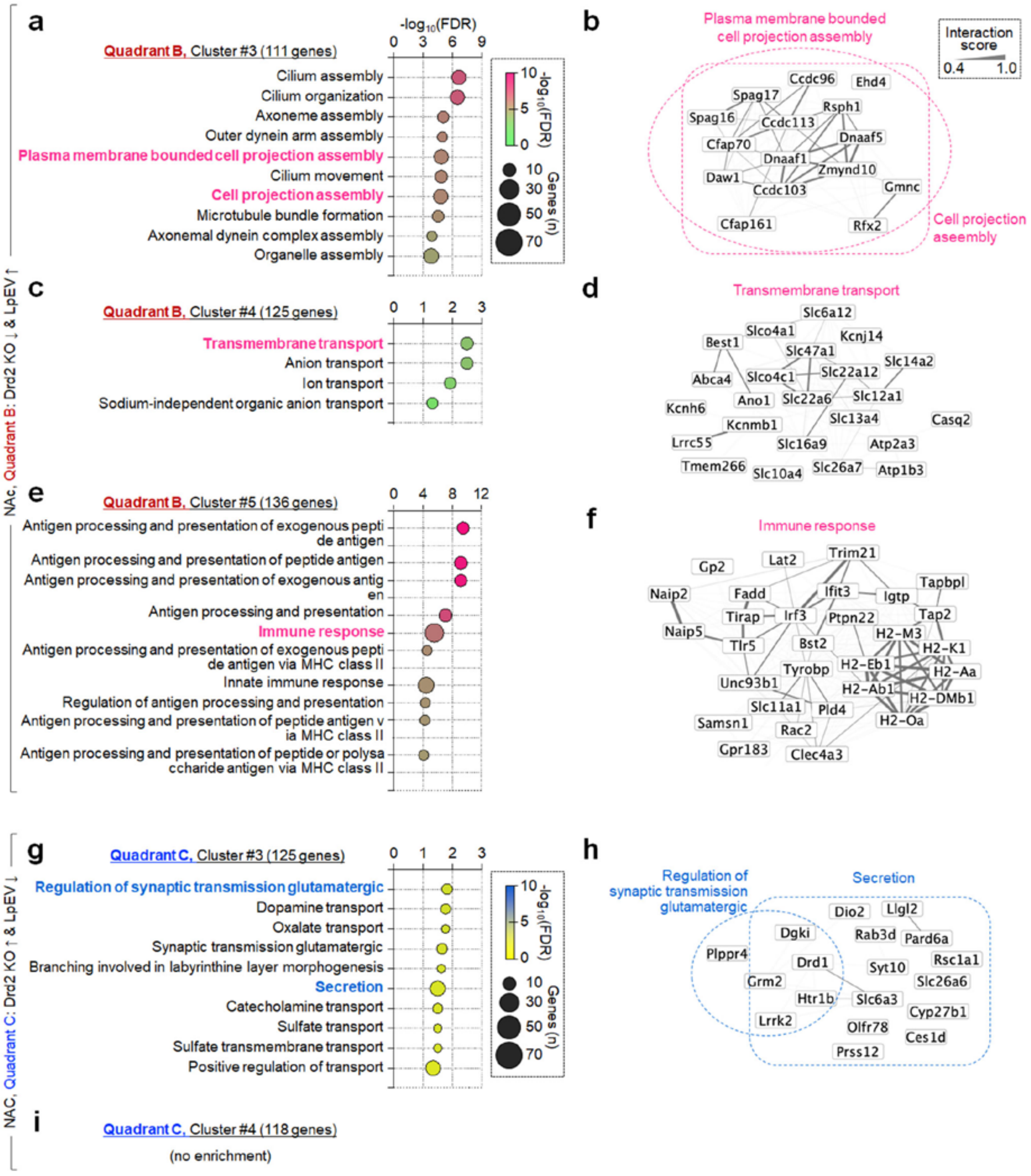

Supplementary Fig. 5. Three clusters in Quadrant B and two clusters in Quadrant C identified in the nucleus accumbens of Drd2 KO mice and Drd2 KO mice treated with LpEV.
a-i Top 10 biological processes in each of the remaining three clusters of genes in Quadrant B (666 genes). Cluster #3 (111 genes) contained genes regulating “cell projection assembly” (**a**), Cluster #4 (125 genes) encompassed genes for “transmembrane transport” (**c**), and Cluster #5 (136 genes) in Quadrant B harbored

genes regulating “immune response” (**e**). The interaction networks of genes within the biological process underlined in red each cluster (**b,d,f**). Clusters #1 and #2 are provided in **Fig. 4f-i**.

g-i Top 10 biological processes in each of the remaining two clusters of genes in Quadrant C (544 genes). Cluster #3 (125 genes) in Quadrant C encompassed genes for “transmembrane transport” (**g**). The interaction network of genes within the selected biological process in blue (**h**). Cluster #4 (118 genes) in Quadrant C did not form a gene group labeled by specific BPs (**i**). Clusters #1 and #2 are provided in **Fig. 4j-m**.

Supplementary Fig. 6

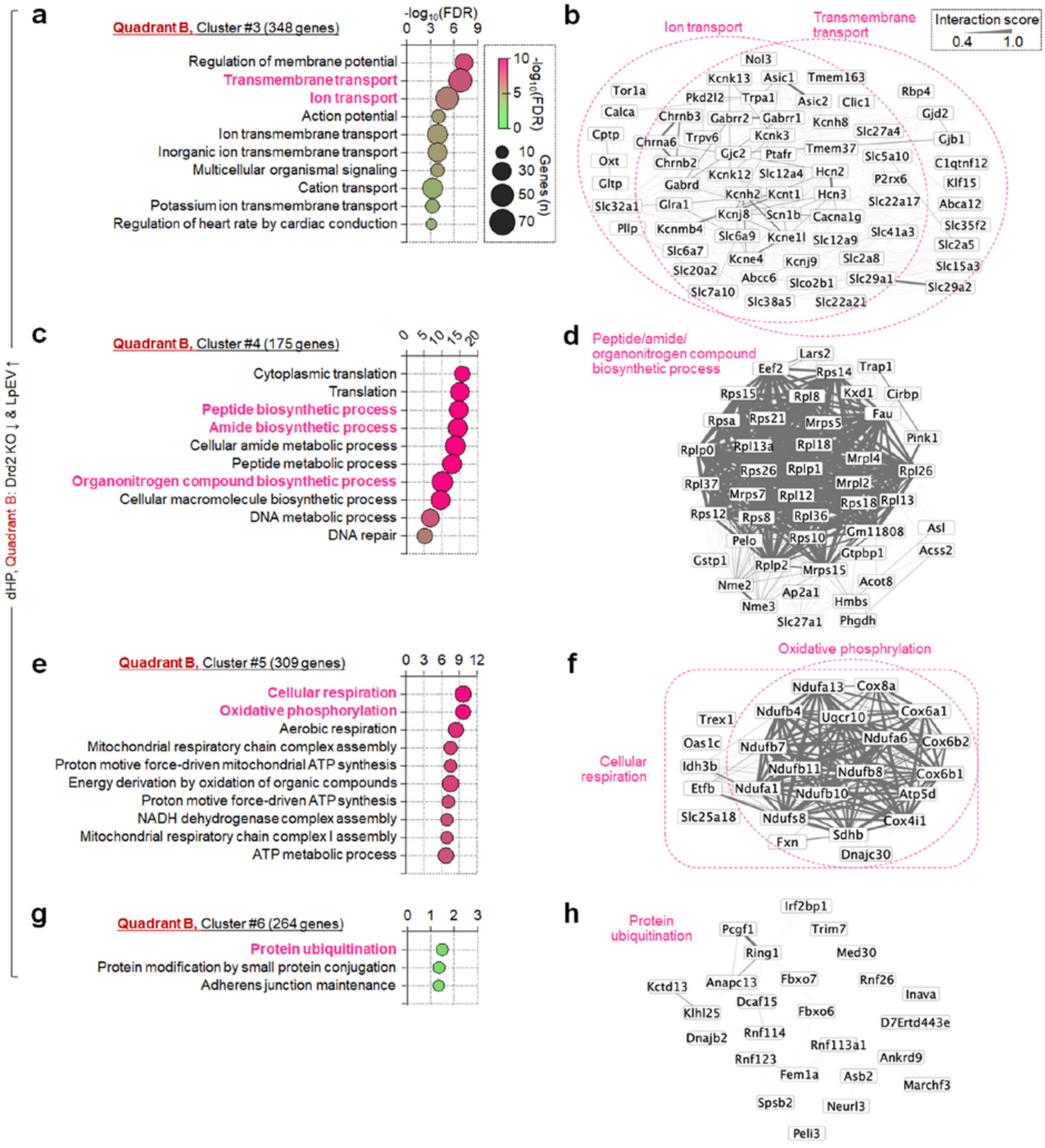

Supplementary Fig. 6. Four clusters in Quadrant B identified in the dorsal hippocampus of Drd2 KO mice and Drd2 KO mice treated with LpEV.
a-h Top 10 biological processes enriched in each of the remaining four clusters of genes identified in Quadrant B. Cluster #3 (348 genes) contained genes regulating "transmembrane transport" (**a**). The interaction network of genes covered by the selected biological process in red (**b**); Cluster #4 (175 genes) encompassed genes involved in peptide, amide, and organic nitrogen compound biosynthesis" (**c**). The

interaction network of genes within the biological process marked in red (**d**); Cluster #5 (309 genes) enriched genes involved in “cellular respiration and oxidative phosphorylation” (**e**). The interaction network of genes within the biological process underlined in red (**f**); and Cluster #6 (264 genes) enriched genes regulating “protein ubiquitination” (**g**). The interaction network of genes within the biological process underlined in red (**h**). Clusters #1 and #2 are presented in **Fig. 5f-i**.

Supplementary Fig. 7

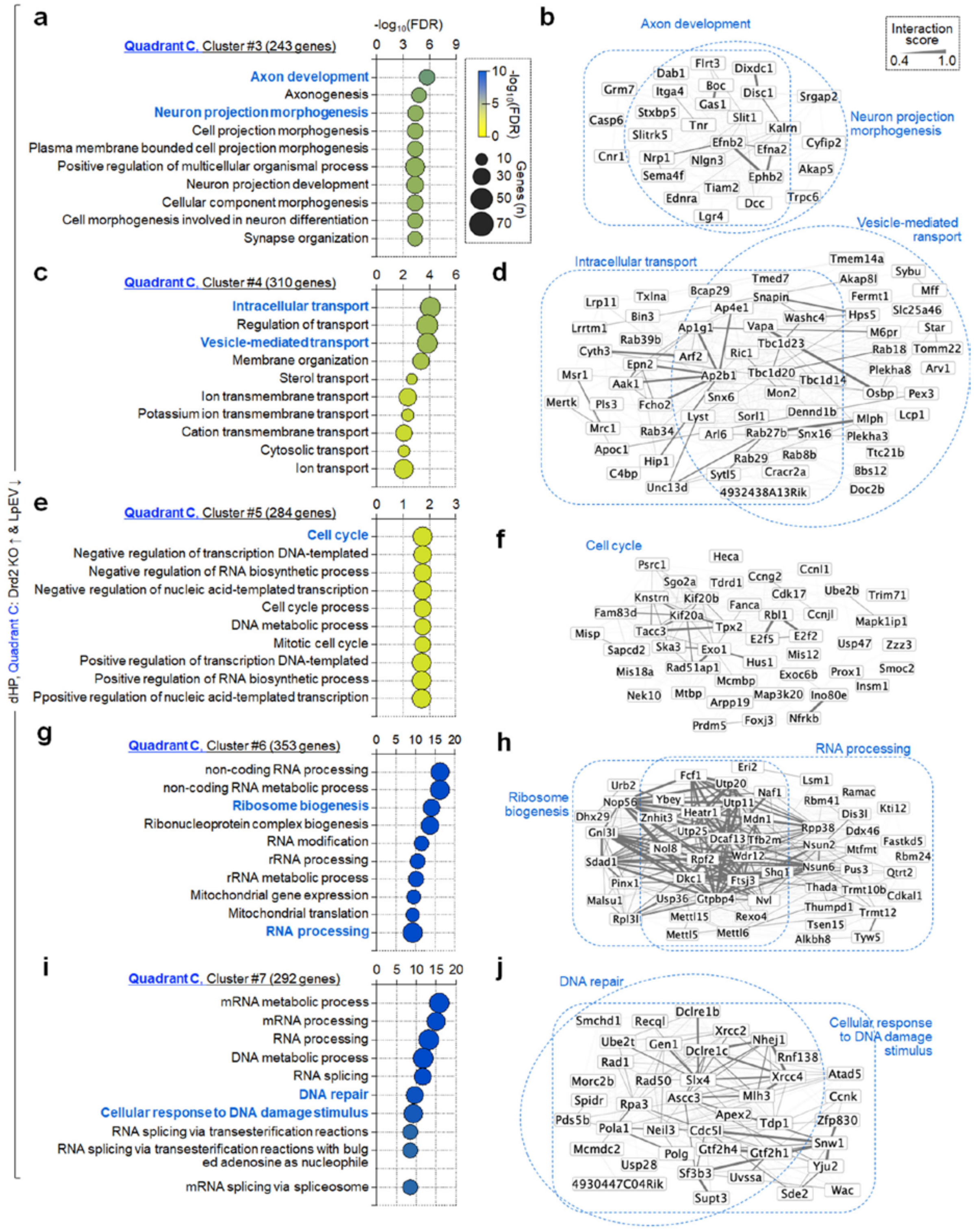

Supplementary Fig. 7. Five clusters in Quadrant C identified in the dorsal hippocampus of Drd2 KO mice and Drd2 KO mice treated with LpEV.

a-j Top 10 biological processes in each of five clusters of genes identified in

Quadrant C. Cluster #3 (243 genes) contained genes regulating “axon development and neuron projection morphogenesis” (**a**). The interaction network of genes within the biological process underlined in blue (**b**); Cluster #4 (310 genes) encompassed genes for “intracellular transport and vesicle-mediated transport” (**c**). The interaction network of genes within the selected biological process in blue (**d**); Cluster #5 (284 genes) harbored genes regulating “cell cycle process” (**e**). The interaction network of genes within the biological process in blue (**f**); Cluster #6 (353 genes) enriched genes involved in ribosome biosynthesis and RNA processing” (**g**). The interaction network of genes within the biological process underlined in blue (**h**); and Cluster #6 (292 genes) enriched genes involved in DNA repair and cellular response to DNA damage stimulus” (**i**). The interaction network of genes within the biological process underlined in blue (**j**). Clusters #1 and #2 are presented in **Fig. 5j-m**.

Supplementary Fig. 8

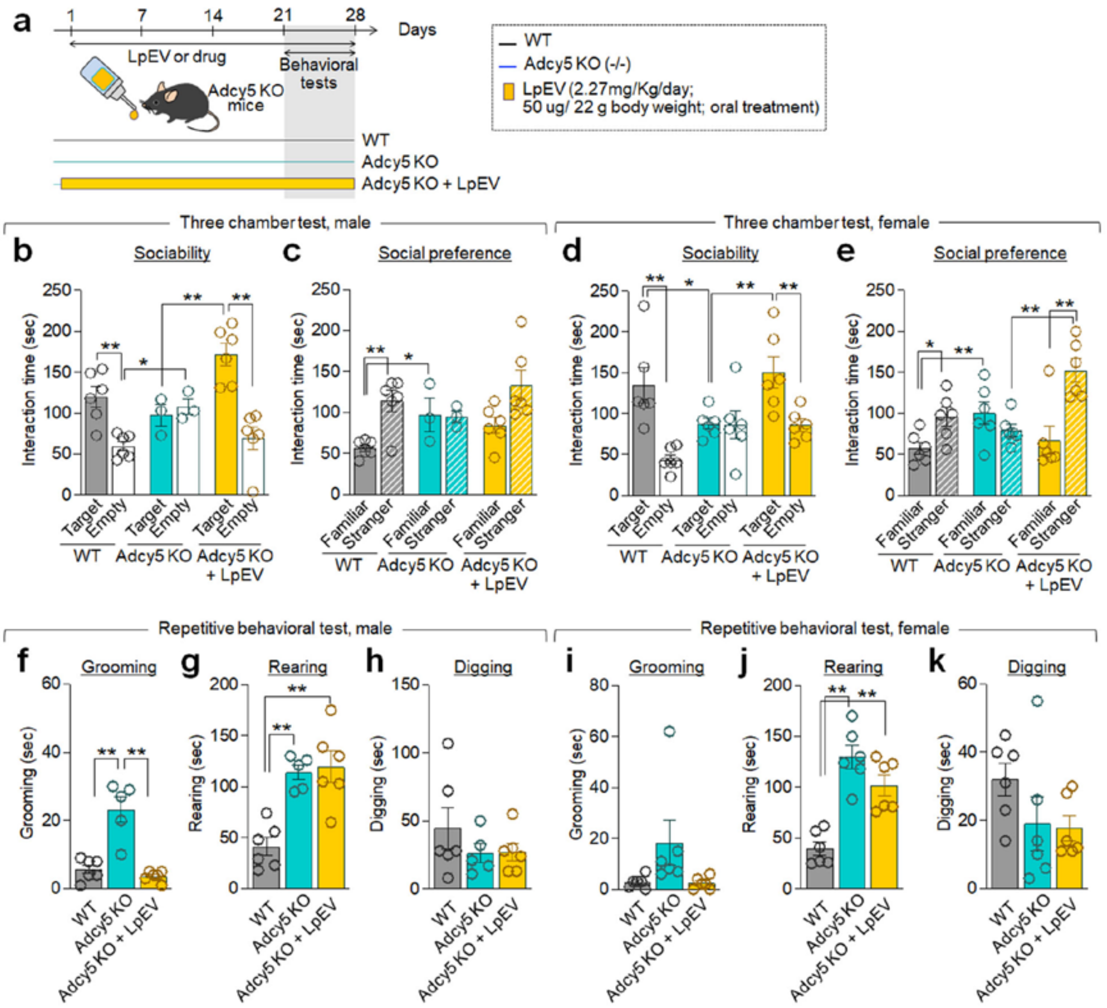

Supplementary Fig. 8. LpEV treatment improved behavioral deficits of Adcy5 KO mice.

a Experimental design. Adcy5 KO mice received LpEV (2.27 mg/Kg/day; 50 μg/ 22 g body weight/day) orally for 3 weeks. This treatment continued until the end of behavioral testing. Behavioral tests were conducted sequentially: Social interaction, social preference, and repetitive tests.

b-e. Social behaviors. Time spent exploring a social target compared to an empty cage (**b,d**) and time spent exploring a novel stranger versus a familiar one (**c,e**) in the three-chamber social behavior test. Data shown for male (**b,c**) and female (**d,e**) Adcy5 KO mice. Male: WT, 6 animals; Adcy5 KO, 3 animals; Adcy5 KO + LpEV, 6 animals. Female: WT, 6 animals; Adcy5 KO, 6 animals; Adcy5 KO + LpEV, 6 animals.

f-k. Repetitive behaviors. Time spent by male (**f,g,h**) and female (**i,j,k**) Adcy5 KO mice on self-directed behaviors of grooming (**f,i**), rearing (**g,j**), and digging (**h,k**).

Male: WT, 6 animals; Adcy5 KO, 3 animals; Adcy5 KO + LpEV, 6 animals. Female: WT, 6 animals; Adcy5 KO, 6 animals; Adcy5 KO + LpEV, 6 animals.

Mean +/- SEM. *, $p < 0.05$; **, $p < 0.01$ (One-way ANOVA, Tukey's post-hoc test; Two-way ANOVA, Fisher's LSD post-hoc test).

Supplementary Fig. 9

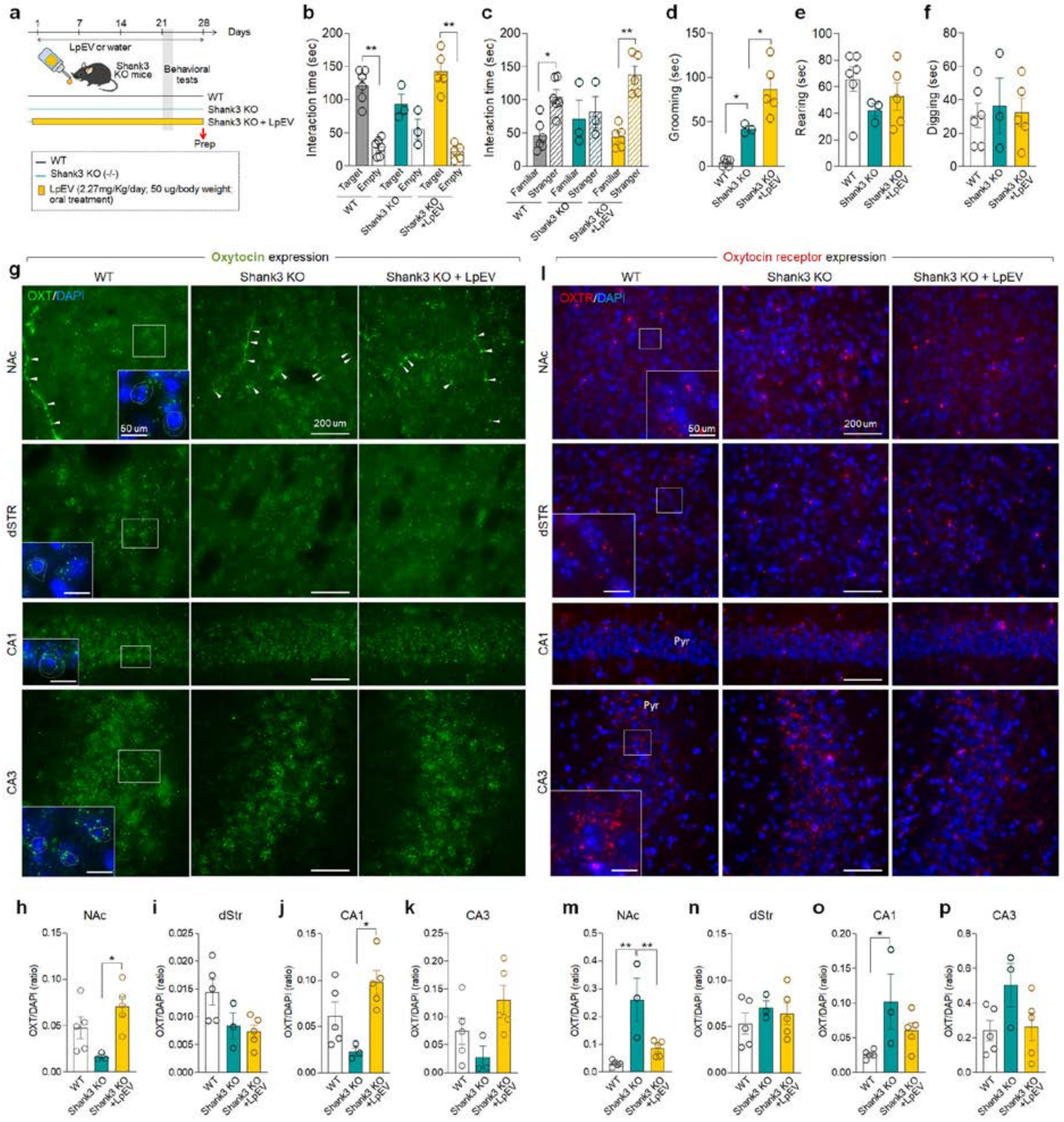

Supplementary Fig. 9. LpEV treatment reversed altered Oxt and Oxtr expression in the brain and alleviated social behavior deficits in Shank3 KO mice

a Experimental design. Shank3 KO mice were administrated with LpEV (2.27 mg/Kg/day; 50 μg/ 22 g body weight/day) orally for 3 weeks. LpEV treatment continued until the end of behavioral testing. Behavioral tests were conducted in the order of social interaction, social preference, and repetitive tests. The experimental groups included 6 wildtype mice (3 males, 3 females), 3 Shank3 KO mice (2 males, 1 female), and 5 Shank3 KO + LpEV mice (3 males, 2 females).

b,c. Social behaviors. Time spent exploring a social target compared to an empty cage (**b**) and time spent exploring a novel stranger versus a familiar one (**c**) in the three-chamber social behavior test. Mean +/- SEM. *, $p < 0.05$; **, $p < 0.01$ (Two-way ANOVA, Fisher's LSD post-hoc test).

d-f. Repetitive behaviors. Time spent on self-directed behaviors of grooming (**d**), rearing (**e**), and digging (**f**) for WT, Shank3 KO mice, and Shank3 KO mice + LpEV groups. Mean +/- SEM. *, $p < 0.05$ (One-way ANOVA, Tukey's post-hoc test).

g-k. Immunobiological staining for Oxt expression (**g**) in the NAc, dStr, CA1 and CA3 regions of the hippocampus of WT, Shank3 KO mice, and Shank3 KO mice + LpEV. Insets, high magnification of the boxed area. Local Oxt expressions appeared as punctuated signals. Arrowheads delineate axon tracts with varicosities of Oxt neurons, likely originating from the hypothalamic Oxt neurons. Note that Shank3 KO mice exhibit unorganized or small varicosities, while those treated with LpEV show more articulated and larger varicosities. These features warrant further investigation. Pyr, pyramid cell layer. Oxt, green; DAPI, blue. Quantification of staining intensity for Oxt (**h,i,j,k**). Oxt signals were quantified as a ratio relative to DAPI staining. WT, 5 animals; Shank3 KO mice, 3 animals; and Shank3 KO mice + LpEV, 5 animals. Mean +/- SEM. *, $p < 0.05$ (One-way ANOVA, Tukey's post-hoc test).

l-p. Immunobiological staining for Oxtr expression (**l**) in the NAc, dStr, CA1 and CA3 region of the hippocampus of WT, Shank3 KO mice, and Shank3 KO mice + LpEV. Oxtr, red; DAPI, blue. Quantification of staining intensity for Oxtr (**m,n,o,p**). Oxtr signals were quantified as a ratio relative to DAPI staining. WT, 5 animals; Shank3 KO mice, 3 animals; and Shank3 KO mice + LpEV, 5 animals. Mean +/- SEM. Mean +/- SEM. *, $p < 0.05$; **, $p < 0.01$ (One-way ANOVA, Tukey's post-hoc test).

Supplementary Materials and Methods

Animals

C57BL/6 mice were purchased from Daehan BioLink (Eumsung, Chungbuk, Republic of Korea). Drd2 KO mice (B6.129S2-Drd2^{tm1Low}/J) and Adcy5 KO mice (B6.129/SvJ-AC5^{tm1Han}/Ewha) were described previously[1,2,3]. Drd2 KO mice and Adcy5 KO mice were backcrossed to C57BL/6 mice for more than 20 generations. Shank3 KO mice (B6.129-Shank3^{tm2Gfng}/J)[4] were obtained from the Jackson laboratory (Bar Harbor, USA), and backcrossed to C57BL/6 mice. For genotyping, the following primer sets were used: 5'-TGATGACTGGGAATGTTGGTGTGC-3' and, 5'-CTCCCCAGAGTTGTGGCAAAAGG-3' for WT (221 bp); and 5'-TGATGACTGGGA ATGTTGGTGTGC-3' and 5'-AGGATTGGGAAGACAATAGCA G-3' ′ for Drd2 KO (329 bp). 5′-GTCCGAGGATGGAGGCAGTT-3′ and 5′-CACCACTGCAATGAGCGCATA-3′ for Adcy5 WT (373 bp); 5′-ACCGTCGAGGATGGAGACGG-3′ and 5′-TGTCCATCTGCTGCACGAGACTA-3′ for Adcy5 KO (514 bp). 5′-GTCCGAGGATGGAGGCAGTT-3′ and 5′-CACCACTGCAATGAGCGCATA-3′ for Adcy5 WT (373 bp); 5′-ACCGTCGAGGATGGAGACGG-3′ and 5′-TGTCCATCTGCTGCACGAGACTA-3′ for Adcy5 KO (514 bp). 5'-GAGACTGATCAGCGCAGTTG-3' and 5'- TGACATAATCGCTGGCAAAG-3' for WT (374 bp); 5'-GAGACTGATCAGCGCAGTTG-3' and 5'-GCTATACGAAGTTATGTCGACTAGG-3' for Shank3 KO (153 bp).

Oxytocin receptor KO mice were generated by genetically crossing a mouse line carrying two loxP sites flanking the Oxtr coding sequence (exons 2 and 3) (Oxtr^{flox}) (Jax strain #008471)[5] with a transgenic line expressing Cre recombinase driven by the EIIa promoter (Jax strain #003724). Successful inactivation of the Oxtr was confirmed by genomic PCR using a forward primer (Seq 1) 5'-ACCCCAGGAAGATGTACCCGTAGTAAAGC-3' and a reverse primer (Seq 4) 5'-TTAGGTCCCAGGAAAGAGTCAGCCGCTCTGCCTGCAGAGAGG-3' for WT and Floxed locus (221 bo for WT and 291 bp for Floxed); and a forward primer (Seq 1) 5'-ACCCCAGGAAGATGTACCCGTAGTAAAGC-3' and a reverse primer (DTAo 10.3) 5'-TGGGAGTCCAGAGATAGTGGAA-3' for the recombinated locus (150 bp).

Homozygous Drd2, Adcy5, and Shank3 knockout mice were generated by crossing heterozygous breeding pairs. Due to developmental lethality and potential C57BL/6 background effects, the observed wildtype-to-knockout ratio was consistently 1:0.5 or lower. Homozygous oxytocin receptor knockout mice rarely survived to adulthood.

After weaning, male and female mice were separately housed with 3-4 animals in regular plastic cages with chopped wood particles (TAPVEI, Paekna, Estonia) to 3 cm from the cage floor in a temperature (22–23 °C)-and humidity (50–60%)-controlled specific-pathogen-free environment under a normal light-dark cycle (light on 7:00 AM). All animals were fed lab chow (Purina Inc., Yongin, Gyeonggi-do, Republic of Korea) and water *ad libitum*.

All mice were handled in accordance with the Guidelines of Animal Care at Ewha Womans University through permission of EWU-IACUC (No. 19-015).

RNA seq analysis

Brain tissue samples were homogenized in Trizol® reagent (#15596018; Invitrogen, CA, USA) by following a manufacture's protocol. Total RNA was isolated using the RNeasy MinElute Cleanup kit (#74204; Qiagen, Hilden, Germany), and then treated with DNase I (New England Biolabs, MA, USA).

RNA sequencing and quality control processes were performed by Macrogen Inc. (Seoul, Republic of Korea). All RNA samples were quantified by Quant-iT RiboGreen RNA Assay Kit (#R11490; Invitrogen, CA, USA) using Victor Nivo Multimode Microplate Reader (#HH350005500; PerkinElmer, Waltham, MA, USA). RNA quality was measured using 2100 Bioanalyzer (#G2939BA; Agillent, CA, USA), and only high-quality RNA samples (400 ng), with 28S/18S rRNA (1.8~2.0) and an RNA integrity number value over 7.0, were used for preparing sequencing libraries.

Purified RNA was randomly fragmented and cDNA was synthesized. After adding different adaptors to both ends of the synthesized cDNA fragments, they were amplified by PCR to an amount sufficient for sequencing using a NovaSeq 6000 platform (Illumina). Sequencing libraries were prepared using the TruSeq™ stranded mRNA kit (#20020594; Illumina Inc. CA, USA). The sequencing libraries were subjected to paired-ends sequencing (101 bp reads per sample) on the HiSeq. 2000 system (Illumina Inc., CA, USA).

Quality of the demultiplexed reads was measured using FastQC. It was conformed that over 95% of trimmed data reached Q30. Trimmed reads were mapped to the *Mus musculus* reference genome (GRCm38.p6) using HISAT2, and were assembled into full-length transcripts using StringTie.

Analyses of differentially expressed genes and gene ontology enrichment

Differentially expressed genes (DEGs) were identified by analyzing mapped read counts from each transcript using edgeR package. All data were normalized using the trimmed mean of M-values method with the '*calcNormFactors'* function from edgeR. Genes with low read counts were filtered out using the filterByExpr function, and only genes with at least 2 reads in two or more samples were considered. The glmQLFTest function is used to perform differential expression analysis with a quasi-likelihood method, and log2-transformed fold-change and its *p*-value were obtained.

RNA seq data of two comparing groups (WT vs Drd2 KO; Drd2 KO vs Drd2 KO+ LpEV) were ranked by signed log10-transformed p-values and analyzed using the Rank-Rank Hypergeometric Overlap method, as described previously[6,7]. **RNA seq** analysis began with 45,777 transcriptome signals. A total of 20,356 genes in the NAc, 19,908 genes in the dSTR, and 19,900 genes in the dHP were obtained and were further filtered based on the Mouse Genome-Database (http://www.informatics.jax.org). They were then analyzed **using the RRHO web application tool** (http://systems.crump.ucla.edu/rankrank). We selected genes whose expression were up- or down-regulated in Drd2 KO mice and their altered expression was reversed by LpEV were selected. These categories of genes were located within Quadrants B and C of the RRHO map. For the dStr and NAc gene

sets, all relevant genes located within Quadrants B and C of the RRHO map were used for further analysis. For the dHP gene sets, the top-60% and 40% of the relevant genes within Quadrants B and C, respectively, of the RRHO map were used for further analysis.

The identified genes in each brain region were grouped into clusters with functionally similar genes using **k-means clustering followed by GO** enrichment using the STRING database (version 12.0; http://string-db.org)[8]. We employed an iterative approach to determine the optimal number of clusters (k), as described prevously[7]. We selected k based on the following three criteria: i) stable enrichment of relevant biological process (BP) terms within clusters, ii) minimal presence or absence of irrelevant BP terms within a cluster, and iii) absence of highly similar BP terms across different clusters. Gene Ontology (GO) enrichment analysis was then performed on each cluster using ShinyGO v0.80 (http://bioinformatics.sdstate.edu/go/)[9] to confirm these conditions. For the analysis, we used the mouse genome assembly GRCm39 and applied a False Discovery Rate (FDR) cutoff of < 0.05 for enriched terms, along with a pathway size between 2 and 2,000 genes. To prioritize the most functionally relevant terms, we only considered the top 20 enriched BP pathways for interpreting the function of each cluster.

Quantitative real-time PCR
Quantitative real-time PCR (qPCR) was carried out as described previously[2,7]. Briefly, brain tissues were homogenized in TRI-zol solution using pellet pestles (Z359971, Sigma-Aldrich, Saint Louis, MO, USA), and total RNA was isolated. Two µg of total RNA was converted into cDNA using a reverse transcriptase system (Promega, Madison, WI, USA). Typically, a qPCR reaction was prepared by combining 4 µl of 1/8 diluted cDNA, 10 µl of 2X iQTM SYBR Green Supermix (Bio-Rad Laboratories, Foster City, CA, USA), 1 µl each of 5 pmol/µl forward and reverse primers, and distilled water in final 20 µl reaction. The qPCR reactions were run on a CFX 96 Real-Time PCR System Detector (Bio-Rad Laboratories) and transcript levels were adjusted using Gapdh.

The primer sets for qPCR analyses are as follows: *Disc1*, forward 5’-CACAGCGGGTTCTGGGATCG-3’ and reverse 5’-TTGCCCACCAGTGAGGCTTG-3’; *Cnr1*, forward 5’-AGCCACGCAGTTCGCATGAT-3’ and reverse 5’-AGCCTAATGTCCATGCGGGC-3’; *Ppp1r1b* (DARPP-32), forward 5’-GAGAGGGGCACCACCCAAAG-3’ and reverse 5’-TCTTCCTCCGAGGCCTGGTT-3’; *Ppp1r9b*, forward 5’-ACTTGGTGGATGTGAGCGCC-3’ and reverse 5’-ACACCCTGACTCCGGCTCAT-3’; *Oxt*, forward 5’-GCCTGCTTGGCTTACTGGCT-3’ and reverse 5’-GGTCCGAAGCAGCGTCCTTT-3’; *Oxtr*, forward 5’-TCAACAGCTGCTGCAACCCA-3’ and reverse 5’-TGCCCTTCAGGTACCGAGCA-3’; *Avp*, forward 5’-CCGAGTGCCACGACGGTTTT-3’ and reverse 5’-GCAGAATCCACGGACTCCCG-3’; *Nr4a3*, forward 5’-CCGCCTTCTACACCGACCAC-3’ and reverse 5’-GCCTTCATCTGCGGGTCCAG-3’. The primers were designed using NCBI Primer-BLAST (www.ncbi.nlm.nih.gov/tools/primer-blast; ref. 10).

Preparation of EVs from *Lactobacillus paracasei* culture.

Lactobacillus paracasei culture and EV preparation were described previously[7,11]. In brief, a *Lactobacillus paracasei* strain, previously isolated from the human body and characterized genomically[11], was used in this study. *Lactobacillus paracasei* was cultured in MRS broth (MB Cell, Seoul, Republic of Korea) at 37°C with a gentle shaking (150 rpm) until the optical density (OD) of 1.0 to 1.5 at 600 nm. The culture was centrifugated at 10,000× **g** at 4°C for 20 min and the supernatant containing EVs was collected, and then passed through a 0.22-μm bottle-top filter (Corning, NY, USA) to remove remaining cells or cell debris. The filtrate was concentrated using a MasterFlex pump system (Cole-Parmer, IL, USA) and a 100-kDa Pellicon 2 Cassette filter (Merck Millipore, MA, USA) with a nominal molecular weight limit (NMWL) of 100 kDa. The concentrated filtrate was then passed through a 0.22-μm bottle-top filter again. The resulting filtrate was centrifugated at 150,000 × *g* for 3 h at 4°C. The pellets containing EVs were collected, washed and resuspended in PBS (137 mM NaCl, 2.7 mM KCl, 10 mM Na_2HPO_4, and 1.8 mM KH_2PO_4). EV protein concentration was determined using a BCA protein assay kit (Thermo Fisher Scientific, MA, USA). EV particle size and numbers in prepared samples were analyzed using a dynamic light scattering measurement system (Zetasizer Nano ZS; Malvern Panalytical, Malvern, UK) and nanoparticle tracking analysis (ZetaView; Particle Metrix GmbH, Ammersee, Germany). EVs were aliquoted and stored at −80 °C until use. *Lactobacillus* paracasei-derived extracellular vesicles are abbreviated as LpEV or Lpc-EV.

The particle size and size distribution of LpEV were assessed by dynamic light scattering measurements using a Zetasizer Nano ZS (Malvern Instruments, Malvern, UK) and analyzed by Dynamic V6 software. LpEV was counted to have an average diameter of 181 +/- 3.0 nm. The biostability of LpEV was examined by treatment of Raw264.7 cells with LpEV freshly prepared and stored at 20°C and LpEV stored at room temperature (RT, 25°C) for 28 days. It was confirmed that LpEV stored at RT retains its physical integrity and biological activity for an extended period.

Administration of LpEV and risperidone to mice

Mice were administered LpEV as described previously[12,13]. Mice aged between 12 weeks and 16 weeks were housed in pairs with littermates, whenever possible, and orally administered LpEV in their drinking water for 4 weeks or indicated time at a dose of 2.27 mg/Kg/day (equivalent to 1.753 x 10^12 particles/Kg/day, or 50 μg/ 22 g body weight/day). **The drinking water contained LpEV at a concentration of 16.7 μg/mL (1.29 x 10^9 EV particles/mL). Fresh LpEV solution was provided by replacing the water bottle every other day. Water consumption was monitored daily to ensure consistent LpEV intake.** The EV concentration in drinking water was adjusted daily, based on the animals' body weight and prior water consumption, to maintain a target dosage of 2.27 mg/Kg/day with a variation of less than 5%. Wildtype littermates and control knockout mice were prepared in parallel in the same room and space, but were treated with drinking water only on the same schedule as the experimental group. Behavioral tests were performed after week 3, and EV administration continued **until the completion of behavioral tests, and then** the animals were sacrificed.

Risperidone was administered to mice as described previously[14] with a minor

modification. **Briefly, mice received risperidone** (#2865; Tocris, Bristol, UK) at a dose of 0.2 mg/Kg/day **via intraperitoneal injection (i.p.) for 7 days, continuing until the completion of behavioral testing 1 hour before every test.**

Whole body imaging and assessment of the distribution of ^{111}In-labeled LpEV
LpEV labeled with a radioactive isotope Indium-111 (^{111}In) was orally administered to mice and its distribution, absorption, and excretion were tracked through the body using SPECT/CT and a gamma counter. All these experiments were carried out by the preclinical research team, Biomedical Research Institute, Seoul National University Hospital. Briefly, LpEV were labeled with ^{111}In via using a crosslinker, NOTA-PEG$_3$-N$_3$, creating ^{111}In-NOTA-PEG$_3$-N$_3$-LpEV. Mice were given ^{111}In alone, ^{111}In-NOTA-PEG$_3$-N$_3$, or ^{111}In-NOTA-PEG$_3$-N$_3$-LpEV. To visualize the biodistribution of the radioactive materials, SPECT/CT imaging was performed at 1 h and 24 h for ^{111}In and ^{111}In-NOTA-PEG$_3$-N$_3$ and at 0.5 h, 1 h, 5 h, 24 h, and 48 h for ^{111}In-NOTA-PEG$_3$-N$_3$-LpEV. The radioactivity signals were also measured using a gamma counter for various organs and tissues.

Immunohistochemical analysis
Immunohistological analysis was performed as previously described[15]. Briefly, mice were anesthetized with 2.5% (w/v) avertin at a dose of 20 μL/g body weight. Mouse brains were perfused with 4% paraformaldehyde via the trans-cardiac method. Brains were removed and post-fixed further in the same solution overnight at 4°C, and coronally cut into 40-μm-thick sections using a vibratome (#VT1000S; Leica Instruments, Nussloch, Germany).

Sections were stored in a cryo buffer containing 30% glycerol at -20°C until use. After washing in PBS, sections were incubated with 5% bovine serum albumin in PBS containing 0.1% Triton X-100 (PBST) for 1 h, and then, reacted with primary antibody at 4°C overnight. After washing, they were incubated with secondary antibody diluted 1:200 in PBST. Anti-Oxytocin antibody (1:100) (#20068, ImmunoStar; Hudson, Wisconsin, USA) and anti-Oxytocin receptor antibody (1:300) (#sc-515809, Santa Cruz Biotech, Dalla, Texas, USA), goat anti-rabbit DyLight™ 488 (#DI-1488, Vector laboratory, Newark, CA, USA) and horse anti-mouse DyLight™ 594 (#DI-2594, Vector laboratory) were used. Stained sections were mounted with mounting solution containing DAPI (#H-1200; Vector Laboratories). Stained images were analyzed using an Olympus BX 51 microscope equipped with a DP71 camera and MetaMorph® Microscopy Automation & Image Analysis software (Molecular Devices, Sunnyvale, CA, USA).

Behavioral tests
Behavioral tests were performed as described previously[1,2]. Mouse activity during each test was recorded using either a video-tracking system (SMART, Panlab Harvard Bioscience, Holliston, MA, USA) or a webcam system (HD Webcam C210, Logitech, Newark, CA, USA). **The behavioral testing room was maintained at 20 lx. White noise (65 dB) provided a masking effect for background noise. All behavior testing equipment was frequently cleaned with 70% ethanol.**

Sociability and social novelty preference tests

The sociability test and social novelty preference test were carried out as described previously[1,2]. The three-chamber apparatus consisted of a rectangular box made of clear polycarbonate, divided into three chambers (each 22 x 32 cm^2) by two transparent walls. Each wall had a retractable doorway (10 cm wide) in the center to allow the mouse access to all chambers.

During the habituation phase, a mouse was placed in the middle chamber and allowed to freely explore all three chambers for 10 minutes. The time spent and movement patterns within each chamber were recorded. Following habituation, the mouse was placed back in the center chamber with the doorways closed. A circular wire cage (12 cm diameter) containing a novel mouse (stranger 1, a naïve C57BL/6, same sex and age) was placed in one side chamber, while an empty cage was placed in the other. The doorways were then opened, and the mouse was allowed to freely explore both chambers for 10 minutes. The time spent and movement traces within each chamber, as well as the time spent sniffing the stranger mouse, were recorded. This phase was regarded as the sociability test. **Following the initial 10-minute sociability test, a novel stranger mouse (stranger 2, another naive C57BL/6, same sex and age) was placed in the previously empty wire cage. The subject mouse was then allowed to explore both chambers for another 10 minutes. During this phase, the time spent sniffing** or directed social interaction **with each stranger and the movement traces within the chambers were recorded. Sniffing was defined as the subject mouse orienting its head towards the wire cage and coming within 2 cm of the grid.**

Repetitive Behavior Assessments

Repetitive behaviors were assessed as described previously[1,2]. In brief, **mice were placed individually in a clean plastic cage with fresh bedding and allowed to habituate for 10 minutes. Following habituation, the time spent in grooming, rearing, and digging was measured for 10 min.** Grooming was defined as the act of animals using their forelimbs to rub their face, body, or head for cleaning or maintenance purposes. Rearing was defined as standing on the hind legs with forelimbs extended against the cage wall or in the air. **Digging was defined as behaviors of using forelimbs or hind limbs to scratch or displace bedding material.**

Statistical analysis

Two-sample comparison was conducted using Student's *t*-test, and multiple comparisons were performed using one-way ANOVA followed by the Newman-Keuls *post hoc* test, two-way ANOVA followed by the Bonferroni *post hoc* test using Graphpad Prism 6 (San Diego, CA, USA). All data are presented as mean ± SEM, and statistical significance was accepted at the 5% level.

References

1. Lee, Y. et al. Excessive D1 dopamine receptor activation in the dorsal striatum promotes autistic-like behaviors. *Mol Neurobiol*. **55**, 5658-5671 (2018).

2. Kim, H. et al. Loss of adenylyl cyclase type-5 in the dorsal striatum produces

autistic-like behaviors. *Mol Neurobiol*. **54**, 7994-8008 (2017).

3. Kelly, M.A. et al. Pituitary lactotroph hyperplasia and chronic hyperprolactinemia in dopamine D2 receptor-deficient mice. *Neuron* **19**, 103–113 (1997).

4. Peça, J. et al. Shank3 mutant mice display autistic-like behaviours and striatal dysfunction. *Nature* **472**, 437-442 (2011).

5. Lee, H.J. et al. A conditional knockout mouse line of the oxytocin receptor. A Conditional Knockout Mouse Line of the Oxytocin Receptor. *Endocrinology* **149**, 3256–3263 (2008).

6. Plaisier, S.B., Taschereau, R., Wong, J.A., & Graeber, T.G. Rank-rank hypergeometric overlap: identification of statistically significant overlap between gene-expression signatures. *Nucleic Acids Res*. **38**, e169 (2010).

7. Kwon, H. et al. Lactobacillus-derived extracellular vesicles counteract Aβ42-induced abnormal transcriptional changes through the upregulation of MeCP2 and Sirt1 and improve Aβ pathology in Tg-APP/PS1 mice. *Exp Mol Med*. **55**, 2067–2082 (2023)

8. Szklarczyk, D. et al. The STRING database in 2023: protein-protein association networks and functional enrichment analyses for any sequenced genome of interest. *Nucleic Acids Res* **51,** D638-D646 (2023).

9. Ge, S.X., Jung, D., Yao, R., & ShinyGO: a graphical gene-set enrichment tool for animals and plants. *Bioinformatics* **36**, 2628–29 (2020).

10. Jian, Y., George, C., Irena Z., Ioana C., Steve, R., & Thomas, L.M. Primer-BLAST: a tool to design target-specific primers for polymerase chain reaction. BMC Bioinformatics 18:13:134 (2012).

11. Choi, J.H. et al. (2020) Lactobacillus paracasei-derived extracellular vesicles attenuate the intestinal inflammatory response by augmenting the endoplasmic reticulum stress pathway. *Exp Mol Med*. **52**, 423–437 (2020).

12. Choi, J., Kim, Y.K., & Han, P.L. (2019). Extracellular Vesicles Derived from **Lactobacillus plantarum** Increase BDNF Expression in Cultured Hippocampal Neurons and Produce Antidepressant-like Effects in Mice. *Exp Neurobiol*. **8**, 158-171.

13. Choi, J., Kwon, H.J., Kim, Y.K., & Han, P.L Extracellular vesicles from Gram-positive and Gram-negative probiotics remediate stress-induced depressive behavior in mice. *Mol. Neurobiol*. **59**, 2715–2728 (2022).

14. Becker JA, et al. Autistic-like syndrome in mu opioid receptor null mice is relieved by facilitated mGluR4 activity. *Neuropsychopharmacology* **39**, 2049-60 (2014).

15. Park, J.Y. et al. Behavioral Engagement with playable objects resolves stress-Induced adaptive changes by reshaping the reward system. *Biol Psychiatry* **91**, 676-689 (2022).
